# Characterization of the virome of *Paracoccus* spp. (*Alphaproteobacteria*) by combined *in silico* and *in vivo* approaches

**DOI:** 10.1038/s41598-019-44460-4

**Published:** 2019-05-27

**Authors:** Przemyslaw Decewicz, Lukasz Dziewit, Piotr Golec, Patrycja Kozlowska, Dariusz Bartosik, Monika Radlinska

**Affiliations:** 1 University of Warsaw, Faculty of Biology, Institute of Microbiology, Department of Bacterial Genetics, Miecznikowa 1, 02-096 Warsaw, Poland; 2 University of Warsaw, Faculty of Biology, Institute of Microbiology, Department of Virology, Miecznikowa 1, 02-096 Warsaw, Poland

**Keywords:** Comparative genomics, Comparative genomics, Bacteriophages, Phage biology, Data mining

## Abstract

Bacteria of the genus *Paracoccus* inhabit various pristine and anthropologically-shaped environments. Many *Paracoccus* spp. have biotechnological value and several are opportunistic human pathogens. Despite extensive knowledge of their metabolic potential and genome architecture, little is known about viruses of *Paracoccus* spp. So far, only three active phages infecting these bacteria have been identified. In this study, 16 *Paracoccus* strains were screened for the presence of active temperate phages, which resulted in the identification of five novel viruses. Mitomycin C-induced prophages were isolated, visualized and their genomes sequenced and thoroughly analyzed, including functional validation of their toxin-antitoxin systems. This led to the identification of the first active *Myoviridae* phage in *Paracoccus* spp. and four novel *Siphoviridae* phages. In addition, another 53 prophages were distinguished *in silico* within genomic sequences of *Paracoccus* spp. available in public databases. Thus, the *Paracoccus* virome was defined as being composed of 66 (pro)phages. Comparative analyses revealed the diversity and mosaicism of the (pro)phage genomes. Moreover, similarity networking analysis highlighted the uniqueness of *Paracoccus* (pro)phages among known bacterial viruses.

## Introduction

*Paracoccus* spp. (*Alphaproteobacteria*) are metabolically versatile bacteria, that have been isolated from a wide range of environments in various geographical locations, e.g.: biofilters for the treatment of waste gases from an animal rendering plant in Germany (*P. alkenifer* DSM 11593), contaminated soil in Japan (*P. aminophilus* JCM 7686 and *P. aminovorans* JCM 7685), rhizospheric soil of an Indian tropical leguminous plant (*P. bengalensis* DSM 17099), sea water from South Korea (P. *haeundaensis* LGM P-21903), marine sediments of the South China Sea (*P. halophilus* JCM 14014T) and marine bryozoan *Bugula plumosa* from North Sea in Germany (*P. seriniphilus* DSM 14827)^1–7^. Some *Paracoccus* spp. have also been recognized as causative agents of human disease^8^. The metabolic flexibility of *Paracoccus* spp. relies mostly on the wide variety of respiratory processes employed by these bacteria, including the usage of nitrate, nitrite, nitrous oxide and nitric oxide as alternative electron acceptors in denitrification, and the ability to use substrates that lack carbon-carbon bonds (e.g. methylamine) as electron donors to respiratory chains^1^.

*Paracoccus* spp. have substantial biotechnological potential, especially in bioremediation, since they can conduct denitrification (e.g. *P. denitrificans*)^9^ and utilize various toxic organic compounds, e.g. *N,N*-dimethylformamide^3^ and herbicides^10^.

*Paracoccus* spp. have multipartite genomes composed of a chromosome plus extrachromosomal replicons, including essential chromids and diverse plasmids^8^. As of June 25^th^ 2018, when data were retrieved for this study, the following DNA sequences had been submitted to NCBI databases: (i) nine complete genomes of *Paracoccus* spp., i.e. *P. denitrificans* PD1222 (GenBank acc. nos. CP000489-CP000491), *P. aminophilus* JCM 7686^11^, *P. aminovorans* JCM 7685^12^, *P. contaminans* RKI 16-01929T^13^, *P. yeei* FDAARGOS_252 (GenBank acc. nos. NZ_CP020440-NZ_CP020447), *P. yeei* TT13^14^, *P. zhejiangensis* J6^15^, *Paracoccus* sp. BM15 (GenBank acc. nos. NZ_CP025408-NZ_CP025411) and *Paracoccus* sp. CBA4604 (GenBank acc. nos. NZ_CP025583-NZ_CP025585), (ii) 54 draft genome sequences, and (iii) 52 plasmids of *Paracoccus* spp.

Although much is known about the metabolic properties and genome architecture of *Paracoccus* spp., there is very little information about phages of these bacteria. To date, only three active phages infecting *Paracoccus* spp. have been identified and described: two lytic phages vB_PmaS-R3 (vB_PmaS_IMEP1)^16^ and Shpa^17^, plus one temperate virus ϕPam-6 of *P. aminophilus* JCM 7686^11^. Moreover, five other prophages (ϕPam-1−ϕPam-5, respectively) were identified within the genome of *P. aminophilus*^11^.

In this study, we identified five novel active temperate phages and 53 prophages in the available genome sequences of *Paracoccus* spp., and performed a thorough comparative analysis of the *Paracoccus* virome.

## Results and Discussion

### Identification and morphology of novel active temperate *Paracoccus* phages

The occurrence of active temperate phages was examined in 16 species of the genus *Paracoccus*: *P. alcaliphilus* JCM 7364, *P. aminovorans* JCM 7685, *P. alkenifer* DSM 11593, *P. bengalensis* DSM 17099, *P. ferroxidans* NCCB 1300066, *P. haeudaensis* LGM P-21903, *P. halophilus* JCM 14014T, *P. homiensis* DSM 17862, *P. kondratievae* NCIMB 13773T, *P. pantotrophus* DSM 11072, *P. seriniphilus* DSM 14827, *P. solventivorans* DSM 11592, *P. sulfuroxidans* JCM 14013, *P. thiocyanatus* JCM 20756, *P. versutus* UW1R and *P. yeei* CCUG 32053. In each case, an exponentially growing culture was exposed to mitomycin C and released phage particles were concentrated using PEG/NaCl solution. This approach resulted in the induction of five phages, named vB_PbeS_Pben1 (*P. bengalensis*), vB_PkoS_Pkon1 (*P. kondratievae*), vB_PsuS_Psul1 (*P. sulfuroxidans*), vB_PthS_Pthi1 (*P. thiocyanatus*) and vBPyeM_Pyei1 (*P. yeei*). It is important to mention, that the term “active” is used in this work for describing mitomycin C-induced and lytic viruses of *Paracoccus* spp., while it is still possible that other, *in silico* distinguished, prophages may respond to another stimuli (e.g. temperature or nutrient deprivation/excess) and therefore they may also be in fact active.

All of the aforementioned strains, plus *P. aminophilus* JCM 7686, were then tested as potential hosts for the induced phages using a spot test. None of the tested strains supported detectable lytic growth of any phage. It was concluded that all of the identified phages are species-specific, with a narrow host range that is possibly confined to their natural host strain.

**Figure 1:**
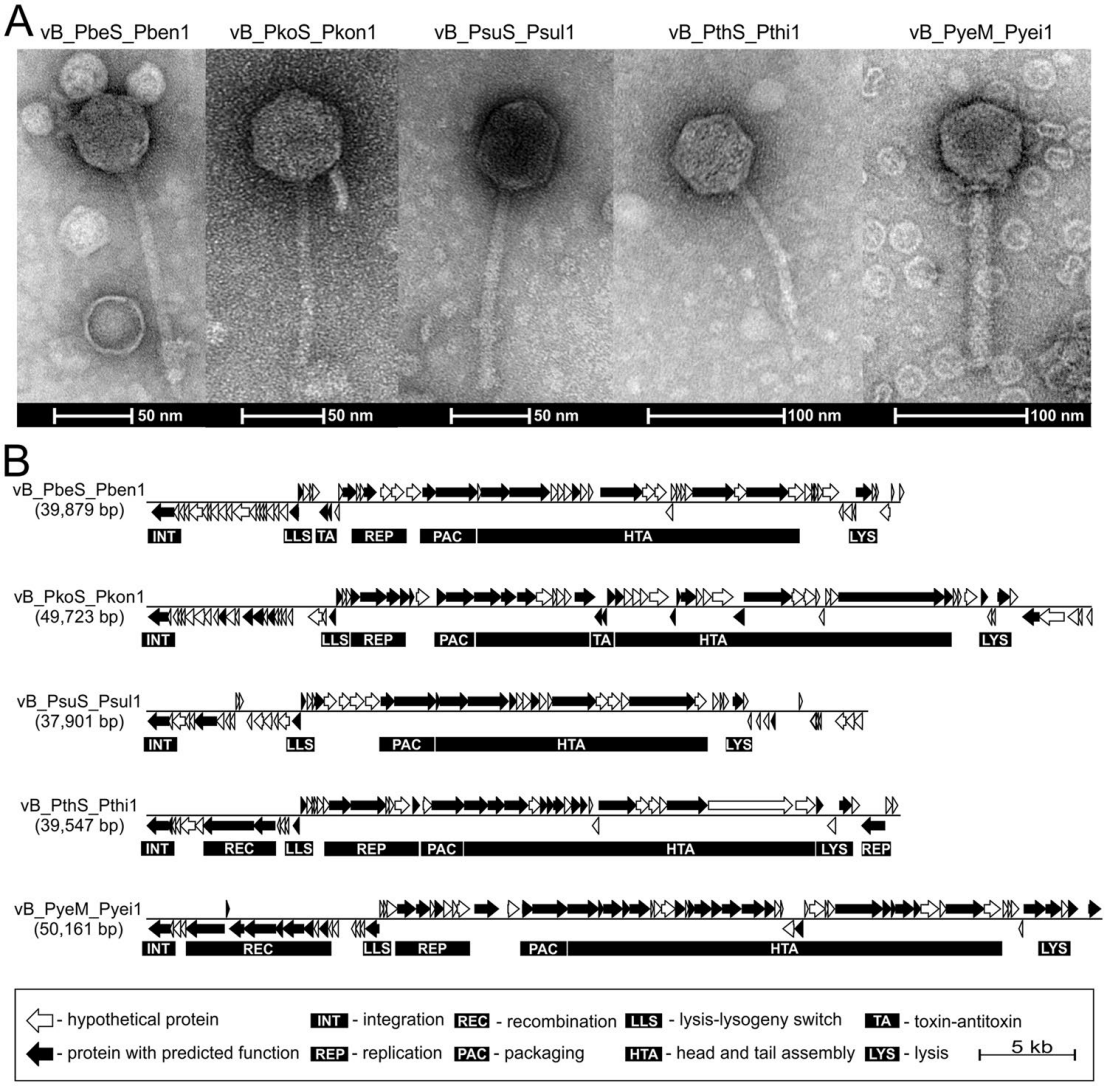
Particle morphology and genome organization of phages vB_PbeS_Pben1, vB_PkoS_Pkon1, vB_PsuS_Psul1, vB_PthS_Pthi1 and vB_PyeM_Pyei1. (**A**) Transmission electron micrographs of the phage particles. A scale bar is shown below each micrograph. (**B**) Phage genome organization. Arrows indicate the transcriptional orientation of the genes. The distinguished genetic modules are indicated by black boxes.

TEM analysis was then performed to visualize virions of the identified phages. This revealed that all the phages have icosahedral heads and tails of the sizes presented in Table 1. The morphological features of these phages indicated that four of them (vB_PbeS_Pben1, vB_PkoS_Pkon1, vB_PsuS_Psul1 and vB_PthS_Pthi1) belong to the *Siphoviridae* family, while vB_PyeM_Pyei1 represents the *Myoviridae* family (Fig. 1A). It is noteworthy that vB_PyeM_Pyei1 is the first representative of the *Myoviridae* family to be identified in *Paracoccus* spp.

**Table 1:** Sizes of heads and tails of the identified *Paracoccus* phages.

Phage	Head width (nm)*	Head length (nm)*	Tail width (nm)*	Tail length (nm)*
vB_PbeS_Pben1	48.2 ± 8.3	49.7 ± 6.9	8.7 ± 0.5	144.0 ± 11.1
vB_PkoS_Pkon1	49.3 ± 1.5	59.2 ± 2.5	8.8 ± 0.3	132.6 ± 9.0
vB_PsuS_Psul1	56.6 ± 4.5	56.3 ± 0.9	8.4 ± 0.4	120.4 ± 5.1
vB_PthS_Pthi1	53.4 ± 2.4	57.7 ± 3.4	7.7 ± 1.1	98.3 ± 8.3
vB_PyeM_Pyei1	58.3 ± 3.0	59.3 ± 2.3	14.3 ± 1.5	135.8 ± 7.8

*The presented sizes are averages (±standard deviation) calculated from measurements of 10 randomly picked phage particles.

### Genomic analysis of identified *Paracoccus* phages

#### General features of active phage genomes

The genomes of the identified active phages were sequenced. After digestion of the *Paracoccus* phage DNAs with the restriction enzymes, no alteration in the banding pattern was observed after heating the DNA to 70 °C (data not shown), which indicated that the ends of their genomes did not form complementary overhangs and the phage DNAs was packaged by a headful mechanism (pac type). The headful mechanism is characteristic for circularly permuted genomes^18^. General characteristics and features of the *Paracoccus* phage genomes are summarized in Table 2.

**Table 2:** General characteristics and features of *Paracoccus* phage genomes.

Phage	Phage Family	Genome size (bp)	GC content (%)	No. of genes	Attachment site	*attB* sequence*
vB_PbeS_Pben1	*Siphoviridae*	39,879	64.3	71	Intergenic region	GCGTCTCGTTTACACTGAGA
vB_PkoS_Pkon1	*Siphoviridae*	49,723	60.6	79	Gene encoding OmpR family transcriptional regulator	GTTTCTCAA(G/C)CAT
vB_PsuS_Psul1	*Siphoviridae*	37,901	60.9	57	tRNA_Trp_(CCA)	CGGTCTCCAAAACCGAGGGTCGTGGGTTCGAGTCCCCCAACCCCTGCCAGT
vB_PthS_Pthi1	*Siphoviridae*	39,547	63.8	52	tRNA_Ser_(GGA)	CCTCACCGTCCGCCA
vB_PyeM_Pyei1	*Myoviridae*	50,161	65.5	75	tRNA_Pro_(TGG)	GACGGTTTTGGGTACCGTAGGCCGGAGGTTCGAATCCTCTCGCCCCGACCAG

*Sequences are shown in the 5′ to 3′ orientation.

Thorough manual sequence annotation of the phage genomes revealed modular structures that are typical for temperate bacteriophages^19^. The distinguished gene clusters determine functions crucial for the phage life cycle, such as integration/excision, DNA recombination, early transcriptional regulation, DNA replication, packaging, capsid and tail assembly, and lysis (Fig. 1B). Specific functions of predicted phage-encoded proteins were assigned on the basis of their similarity to known phage proteins. Features of the distinguished genes are summarized in Supplementary Table S1. Only one putative tRNA gene was identified within the genome of phage vB_PbeS_Pben1 – tRNA_Val_(TAC). No obvious biological function could be attributed to 62% of the predicted phage gene products, so these were assigned as hypothetical proteins.

Although the aforementioned genetic modules with predicted functions show conservation of their order within the analyzed genomes, only two regions of sequence similarity were found in the DNA sequences of phages vB_PbeS_Pben1 and vB_PsuS_Psul1. The first region contains 13 predicted genes encoding proteins (Pben1_p36-p48 and Psul1_p26-p38, respectively) sharing at least 46% amino acid (aa) identity (Supplementary Table S2). Within this region there are genes encoding terminase and phage structural proteins. The second conserved region is shorter and composed of three genes encoding a putative Cro-like protein and two hypothetical proteins (Pben1_p20-p22 and Psul1_p17-p18, respectively) (Supplementary Table S2).

#### Prophage attachment sites and integration modules

In the lysogenic state a prophage is usually flanked by short directly repeated sequences – *attL* and *attR*^20^. To identify putative attachment sites, bacterial genomic sequences (of the hosts from which the phages have been induced) adjacent to the identified prophages were screened for the presence of direct repeats. This analysis revealed that the vB_PsuS_Psul1, vB_PthS_Pthi1 and vB_PyeM_Pyei1 phages integrated at the 3′ ends of tRNA genes [tRNA_Trp_(CCA), tRNA_Ser_(GGA) and tRNA_Pro_(TGG), respectively], and their integration reconstituted an intact copy of the target genes. Introduction of vB_PkoS_Pkon1 into the host chromosome disrupted a gene encoding a putative OmpR transcriptional regulator, while vB_PbeS_Pben1 integrated within an intergenic region between genes encoding a putative oxidoreductase and formyl-CoA transferase (Table 2).

Site-specific recombination between *attB* and *attP* is mediated by the phage integrases, i.e. tyrosine or serine recombinases^20^. The predicted integrases of all analyzed phages belong to the tyrosine recombinase (XerC/XerD) family, but they share little sequence similarity.

#### Lysogeny control regions

The switch between the lytic and lysogenic cycles of temperate phages is dependent on the expression of divergently transcribed genes encoding functional homologues of λ regulatory proteins CI and Cro^21^. In all analyzed *Paracoccus* phages, predicted CI- and Cro-like repressors, belonging to the XRE family of transcriptional regulators (COG2932), were identified (Supplementary Table S1, Fig. 1B). Each regulatory protein contains a helix-turn-helix domain (HTH, pfam01381), but only one CI-like protein, Pyei1_p19 of vB_PyeM_Pyei1, has an S24 signal peptidase domain (pfam00717).

#### Replication modules

DNA segments adjacent to the lysis/lysogeny switch modules of tailed phages usually contain a cluster of genes involved in DNA replication. Genes encoding predicted replication initiation proteins could be distinguished in the genomes of four phages, i.e. vB_PbeS_Pben1 (*pben1_p*3*2*), vB_PkoS_Pkon1 (*pkon1_p28*), vB_PthS_Pthi1 (*pthi1_p19*-*p20*) and vB_PyeM_Pyei1 (*pyei1_p23*) (Table S1, Fig. 1B). None of the proteins encoded by vB_PsuS_Psul1 exhibit sequence similarity to previously described phage replication proteins. However, the Psul1_p21 protein of this phage contains a predicted HTH DNA-binding domain NUMOD1 and we hypothesize that it may be involved in the virus replication process.

#### Toxin-antitoxin systems

Toxin-antitoxin (TA) operons are commonly found within bacterial genomes. They encode two components: a stable toxin, which recognizes a specific cellular target and evokes a bactericidal or bacteriostatic effect, and a labile antitoxin that counteracts the toxin. These loci play important roles in bacterial growth, physiology and pathogenicity. They can also stabilize mobile genetic elements (MGEs) by elimination of MGE-less cells from a bacterial population^22^.

TA systems were identified in two prophages – vB_PbeS_Pben1 (*pben1_p24-p25)* and vB_PkoS_Pkon1 (*pkon1_p43*-*p44*). In both cases the TA system genes are oriented oppositely to the surrounding genes. The *pben1_p24-p25* locus encodes a HicA-type toxin (Pben1_p25; COG1724), possibly involved in mRNA cleavage^23^, while *pkon1_p43*-*p44* encodes an mRNA-degrading toxin of the RelE/ParE family (Pkon1_p44; COG2026)^24^.

We tested the functionality of these systems in a heterologous host – *P. versutus* UW225. For this purpose, both TA systems were cloned into the stability test vector pABW3, yielding pABW3-TA_PBE (TA of vB_PbeS_Pben1) and pABW3-TA_PKO (TA of vB_PkoS_Pkon1). Plasmid pABW3-TA_PBE was stably maintained in strain UW225 (no plasmid-less cells were detected after 30 generations of growth under non-selective conditions), but this was not the case for pABW3-TA_PKO (9% of cells carried the plasmid following growth without selection). The “empty” vector pABW3 was present in 4% of cells after the same period of non-selective growth. These results indicate that *pben1_p24-p25* comprises an active stabilizing system, while *pkon1_p43*-*p44* seems to be non-functional, at least in this host. However, this TA system might be active in other hosts, as was previously observed for *tad*-*ata*-type systems^25^.

#### Lysis modules

Many dsDNA bacteriophages use a holin-endolysin system for host cell lysis to release progeny virions^26^. Endolysins are responsible for the degradation of the bacterial cell wall, causing the release of newly formed viral particles. These enzymes are synthesized without a signal sequence and thus accumulate in the cytosol during the viral life cycle^26^. Holins accumulate in the cell membrane and then perforate it, causing lesions that allow endolysin to access the cell wall peptidoglycan^27^.

A BLASTp search indicated that predicted proteins Pben1_p67, Pkon1_p73, Psul1_p45, Pthi1_p48 and Pyei1_p72 (Supplementary Table S1) are endolysins. Pkon1_p73 and Pthi1_p42 share 92% aa identity, while Pben1_p67 and Pyei1_p72 share 74.3% identity. These four proteins were classified as *N*-acetylmuramoyl-L-alanine amidases, i.e. enzymes that cleave the amide bond between *N*-acetylmuramic acid (MurNAc) and the first highly conserved stem L-alanine residue^26^. Psul1_p45, the predicted endolysin of phage vB_PsuS_Psul1, is a glycosidase (or muramidase), that presumably cleaves the linkage between MurNAc and *N*-acetylglucosamine^26^.

The identification of holin-encoding genes within the genomes of *Paracoccus* phages was challenging. Only Pkon1_p70 and Pthi1_p46 (64% aa sequence identity) share significant sequence similarities with known holins. In addition, membrane-spanning domains were detected in Psul1_p43 and Pyei1_p73 using the programs TMHMM and TMPRED. However, confirmation that these proteins are indeed holins will require further experimental study.

#### DNA methyltransferase genes

Lytic and lysogenic phages often encode multi- and monospecific solitary DNA methyltransferases (MTases), not associated with restriction endonucleases^28^. They may also have complete restriction-modification (RM) systems^29^.

Three of the analyzed phages (vB_PbeS_Pben1, vB_PkoS_Pkon1 and vB_PyeM_Pyei1) possess genes encoding orphan DNA MTases. Protein Pyei1_p05 exhibits similarity to several well characterized C5-methylcytosine (m^5^C) MTases, e.g. JCM7686_0772 and JCM7686_2655 of the prophages ФPam-1 and ФPam-5 of *P. aminophilus* JCM768 (~69% aa identity), respectively^11^. It was previously demonstrated that DNA modified by m^5^C MTases homologous to Pyei1_p05 is protected from cleavage by a wide variety of cytosine methylation-sensitive restriction endonucleases^11^. Therefore, it may be assumed that the Pyei1_p05 MTase also has a relaxed substrate specificity. The predicted MTases of the phages vB_PbeS_Pben1 and vB_PkoS_Pkon1 exhibit similarity to N6-adenine (m^6^A) modification enzymes. Protein Pben1_p29 is similar to DNA MTases encoded by viruses infecting *Alphaproteobacteria* (e.g. GenBank acc. no. YP_009146999 of *Aurantimonas* phage AmM-1; 50% aa identity). This group of viral MTases targets a sequence (5′-GANTC-3′) that is also recognized by *Alphaproteobacteria*-specific cell cycle-regulated MTase CcrM^30,31^. The *pben1_p29* gene is located adjacent to the predicted replication module of phage vB_PbeS_Pben1. A putative m^6^A MTase was also identified in phage vB_PkoS_Pkon1. The *pkon1_p75* gene is located at the end of the right arm of this genome (Supplementary Table S1).

#### Auxiliary metabolic genes

Temperate bacteriophages can contain auxiliary genes that modulate and augment host cell metabolism during infection and facilitate production of new viruses. A presumed auxiliary metabolic gene was found only within the genome of vB_PyeM_Pyei1. The *pyei1_p13* gene encodes a homolog of a tellurite-resistance protein TerB. TerB is encoded within a tellurite resistance operon (*terZABCDEF*) found in e.g. *E. coli* APEC O1 plasmid pAPEC-O1-R^32^. Homologous (56.6% aa identity with the Pyei1_p13 protein), TerB protein is encoded by *Sinorhizobium* phage ФM5 (GenBank acc. no. ARV77549).

### Identification and characterization of (pro)phages occurring in *Paracoccus* spp. genomes

*Paracoccus* spp. genomes (nine complete and 54 drafts) and 52 complete plasmid sequences (present in NCBI databases on June 25^th^, 2018) were inspected for the presence of prophage regions using the PhiSpy tool^33^. Obtained results were afterwards manually curated. Only the regions comprising complete prophage genomes were included in further analyses. This was determined based on the presence of phage integration, replication, packaging, structural and lysis modules. Additionally, boarders of prophages were indicated based on the presence of predicted *attB* and *attP* sequences or, when not distinguishable, differences in %GC content between the prophage region and the surrounding host genome. As a result, 53 novel prophages were identified (Supplementary Dataset 1). These were detected in 29 *Paracoccus* strains; thus nearly half of the tested bacteria were lysogens. All of the identified prophages were classified within the order *Caudovirales* using VIRFAM^34^. Fifty were classified as members of the *Siphoviridae*, eight as *Podoviridae* (the first *Podoviridae*-like phages specific to *Paracoccus*) and only one as *Myoviridae* (Table 3). Interestingly, among the 29 lysogenic strains, 14 are polylysogens, carrying multiple prophages within their genomes: 6 strains have two prophages, 3 have three prophages, 3 have four prophages, and single strains have five and six prophages, respectively (Table 3).

**Table 3:** General properties of *Paracoccus* prophages identified in genomic sequences in the NCBI database.

Host	Phage name	Family	Integration strategy*	Integration site	Genome size (bp)	% GC	No. of genes	Genome acc. no (coordinates)
*P. aminophilus* JCM 7686	vB_PamS_Pami1	*Siphoviridae*	tyr	tRNA-Pro(TGG)	43,882	61.6	57	NC_022041 (725,048–768,929)
	vB_PamS_Pami2	*Siphoviridae*	tyr	intergenic	37,658	60.8	51	NC_022041 (1,209,587–1,247,244)
	vB_PamS_Pami3	*Siphoviridae*	tyr	tRNA-Arg(CCG)	35,083	61.9	53	NC_022041 (2,047,014–2,082,096)
	vB_PamS_Pami4	*Siphoviridae*	tyr	partial lyase protein	48,068	60.4	63	NC_022041 (2,286,040–2,334,107)
	vB_PamS_Pami5	*Siphoviridae*	tyr	tRNA-Gly(TCC)	43,256	61.0	59	NC_022041 (2,707,458–2,750,713)
	vB_PamS_Pami6	*Siphoviridae*	ser	tRNA-Met(CAT)	38,779	61.6	57	NC_022041 (3,004,809–3,043,587)
*P. aminovorans* DSM 8537	vB_PamS_PD1	*Siphoviridae*	ser	not identified	46,846	66.9	53	NZ_FOPU01000032 (604–47,449)
*P. aminovorans* HPD-2	vB_PamP_PD2	*Podoviridae*	tyr	tRNA-Thr(CTG)	42,749	63.7	80	NZ_KQ955208 (101,365–144,113)
	vB_PamS_PD3	*Siphoviridae*	ser	not identified	41,904	67.7	48	NZ_KQ955210 (20,997–179,000)
*P. contaminans* RKI	vB_PcoS_PD4	*Siphoviridae*	tyr	tRNA-Met(CAT)	43,069	65.1	47	NZ_CP020612 (535,143–578,211)
	vB_PcoS_PD5	*Siphoviridae*	tyr	tRNA-Met(CAT)	61,208	65.2	54	NZ_CP020612 (1,101,277–1,162,484)
	vB_PcoS_PD6	*Siphoviridae*	ser	not identified	41,102	68.5	50	NZ_CP020612 (2,589,532–2,630,633)
	vB_PcoS_PD7	*Siphoviridae*	ser	not identified	51,188	68.2	58	NZ_CP020612 (2,662,598–2,713,785)
*P. denitrificans* DSM 413	vB_PdeP_PD8	*Podoviridae*	tyr	tRNA-Thr(TGT)	43,858	64.6	73	NZ_FNEA01000026, NZ_FNEA01000018 (58,894–61,814, 1–41,307)
*P. denitrificans* DSM 415	vB_PdeP_PD9	*Podoviridae*	tyr	tRNA-Thr(TGT)	43,779	64.6	73	NZ_FOYK01000026, NZ_FOYK01000018 (58,876–61,788, 1–40,867)
*P. denitrificans* ISTOD1	vB_PdeS_PD10	*Siphoviridae*	tyr	tRNA-Arg(TCT)	41,212	65.1	61	NZ_PPGA01000004 (118,900–160,111)
*P. denitrificans* PD1222	vB_PdeS_PD11	*Siphoviridae*	tyr	tRNA-Ser(GCT)	42,731	64.4	61	NC_008686 (318,321–361,051)
	vB_PdeP_PD12	*Podoviridae*	tyr	tRNA-Thr(TGT)	43,827	64.6	75	NC_008687 (875,986–919,812)
*P. homiensis* DSM 17862	vB_PhoS_PD13	*Siphoviridae*	ser	not identified	44,822	67.7	56	NZ_FOHO01000007 (108,618–153,439)
*P. pantotrophus* DSM 1403	vB_PpaP_PD14	*Podoviridae*	tyr	tRNA-Arg(CTT)	38,850	62.9	74	NZ_FPKI01000005 (21–38,870)
*P. pantotrophus* J46 J46	vB_PpaP_PD15	*Podoviridae*	tyr	putative ompR regulator	42,547	61.9	72	NZ_KI912520 (48,334–90,880)
*P. saliphilus DSM* 18447	vB_PsaS_PD16	*Siphoviridae*	tyr	tRNA-Gly(GCC)	45,275	59.5	55	NZ_FTOU01000011 (482–45,756)
*P. sanguinis* 10990	vB_PsaS_PD17	*Siphoviridae*	tyr	not identified	52,526	64.2	59	NZ_JRKR01000006 (2–52,527)
*P. sanguinis* 39524	vB_PsaS_PD18	*Siphoviridae*	tnp	not identified	41,698	68.5	60	NZ_JRKP01000001 (14,587–56,284)
	vB_PsaS_PD19	*Siphoviridae*	tnp	not identified	39,502	68.6	53	NZ_JRKP01000010 (2–39,503)
*P. sanguinis* 4681	vB_PsaS_PD20	*Siphoviridae*	ser	not identified	41,827	68.4	48	NZ_JRKT01000001 (8,678–50,504)
	vB_PsaS_PD21	*Siphoviridae*	tyr	not identified	38,594	65.3	46	NZ_JRKT01000026 (2–38,595)
*P. sanguinis* 5503	vB_PsaS_PD22	*Siphoviridae*	tyr	tRNA-Phe(GAA)	53,314	66.0	59	NZ_JRKQ01000001 (25,096–78,409)
	vB_PsaS_PD23	*Siphoviridae*	ser	not identified	61,802	66.5	70	NZ_JRKQ01000003 (21,690–83,491)
	vB_PsaS_PD24	*Siphoviridae*	tyr	tRNA-Met(CAT)	49,592	65.1	52	NZ_JRKQ01000004 (242–49,833)
	vB_PsaS_PD25	*Siphoviridae*	tyr	not identified	32,827	66.9	42	NZ_JRKQ01000005 (3,432–36,258)
	vB_PsaS_PD26	*Siphoviridae*	tnp	not identified	44,577	68.5	56	NZ_JRKQ01000008 (2–44,578)
*P. sanguinis* DSM 29303	vB_PsaS_PD27	*Siphoviridae*	tnp	not identified	41,697	68.5	60	NZ_FNNA01000001 (674,710–716,406)
	vB_PsaS_PD28	*Siphoviridae*	tnp	not identified	39,255	68.5	57	NZ_FNNA01000006 (82,874–122,128)
	vB_PsaS_PD29	*Siphoviridae*	tyr	tRNA-Met(CAT)	51,410	65.3	58	NZ_FNNA01000009 (123–51,532)
*P. sediminis* DSM 26170	vB_PseS_PD30	*Siphoviridae*	tyr	tRNA-Met(CAT)	48,532	64.7	48	NZ_FZNM01000001 (93–48,624)
*P. solventivorans* DSM 6637	vB_PsoS_PD31	*Siphoviridae*	ser	not identified	42,946	66.0	63	NZ_FRCK01000001 (414,364–457,309)
*Paracoccus* sp. BM15	vB_PspS_PD32	*Siphoviridae*	tyr	tRNA-Met(CAT)	51,828	60.8	51	NZ_CP025408 (420,882–472,709)
	vB_PspS_PD33	*Siphoviridae*	tyr	tRNA-Met(CAT)	50,035	63.6	58	NZ_CP025408 (1,328,234–1,378,268)
*Paracoccus* sp. CBA4604	vB_PspS_PD34	*Siphoviridae*	ser	not identified	44,369	67.8	53	NZ_CP025583 (807,987–852,355)
	vB_PspS_PD35	*Siphoviridae*	ser	not identified	50,207	63.2	54	NZ_CP025583 (862,881–913,087)
	vB_PspS_PD36	*Siphoviridae*	tyr	not identified	44,896	64.7	53	NZ_CP025583 (43,796–88,691)
*Paracoccus* sp. J39	vB_PspS_PD37	*Siphoviridae*	tyr	*dusA*	40,213	63.6	65	NZ_JAEN01000011 (46,163–86,375)
*Paracoccus* sp. N5	vB_PspS_PD38	*Siphoviridae*	tyr	tRNA-Pro(TGG)	46,553	64.3	66	NZ_AQUO01000001 (2,255,488–2,302,040)
*Paracoccus* sp. S4493	vB_PspS_PD39	*Siphoviridae*	tyr	tRNA-Gly(CCC)	37,866	62.7	55	NZ_JXYF01000001 (25,265–63,130)
	vB_PspS_PD40	*Siphoviridae*	tyr	Intergenic	36,248	63.7	50	NZ_JXYF01000039 (20–36,267)
*Paracoccus* sp. SCN 68–21	vB_PspP_PD41	*Podoviridae*	tyr	tRNA-Thr(GGT)	48,266	64.6	63	MEES01000006 (113,916–162,181)
	vB_PspS_PD42	*Siphoviridae*	tyr	tRNA-Cys(GCA)	42,396	63.7	55	NZ_JPKW01000001 (529,373–571,768)
	vB_PspP_PD43	*Podoviridae*	tyr	tRNA-Lys(CAA)	44,599	63.4	67	NZ_JPKW01000003 (122,849–167,447)
	vB_PspS_PD44	*Siphoviridae*	tyr	tRNA-Gln(TTC)	41,926	63.9	71	NZ_JPKW01000009 (114,600–156,525)
*P. sphaerophysae* HAMBI 3106	vB_PspS_PD45	*Siphoviridae*	tyr	tRNA-Met(CAT)	58,117	66.0	59	NZ_JRKS01000013 (81–58,197)
*P. versutus* DSM 582	vB_PveS_PD46	*Siphoviridae*	ser	not identified	41,696	67.6	49	NZ_JRKO01000007 (57,139–98,834)
*P. yeei* ATCC BAA-599	vB_PyeS_PD47	*Siphoviridae*	tyr	Intergenic	56,744	61.8	59	NZ_KK211402 (25,940–82,683)
	vB_PyeS_PD48	*Siphoviridae*	ser	not identified	43,101	68.1	55	NZ_JHWH01000027 (8,448–51,578)
	vB_PyeS_PD49	*Siphoviridae*	tyr	tRNA-Met(CAT)	50,833	65.3	60	NZ_KK211402 (315,002–365,834)
*P. yeei* TT13	vB_PyeM_PD50	*Myoviridae*	tyr	tRNA-Pro(TGG)	54,200	65.0	80	CP024422 (2,080,701–2,134,900)
	vB_PyeS_PD51	*Siphoviridae*	tyr	tRNA-Asn(GTT)	38,463	61.7	39	CP024422 (2,509,005–2,547,467)
	vB_PyeS_PD52	*Siphoviridae*	ser	not identified	52,363	67.8	61	CP024422 (2,660,340–2,712,702)
	vB_PyeS_PD53	*Siphoviridae*	ser	not identified	44,067	65.3	58	CP024422.1 (2,725,351–2,769,417)

*Names tyr, ser and tnp refer to tyrosine recombinase, serine recombinase and Mu-like transposase, respectively.

The integration modules of the identified prophages encode tyrosine recombinases (38 prophages), serine recombinases (16) or Mu-like transposases (5) (Supplementary Table S3). Putative integration sites were identified for the majority of the tyrosine recombinase-encoding viruses (Supplementary Table S3). For 31 prophages these sites were various tRNA genes of which the most commonly targeted were (i) tRNA_Met_(CAT) used by 10 prophages and (ii) tRNA_Pro_(TGG) by four prophages, including active phage vB_PyeM_Pye1. These observations corroborate previous findings regarding the preferential integration of phages (and other integrative elements) within tRNA genes^35^.

With regard to phage structural proteins, the presence of the coding sequence for a nearly 700-amino acid-long protein in 17 (34%) of the *Siphoviridae* prophages (indicated as fused in Supplementary Table S4) is noteworthy. The best BLASTp hits for these proteins are annotated as peptidases of the U35 or U37 families, but characteristic domains for these could not be identified. Instead, a caseinolytic protease domain (ClpP; peptidase S14) cd07016 was always present in the N-terminal region. In addition, a Mu-like prophage major head subunit gpT domain (pfam10124) was identified in the C-terminal region of all these proteins. These observations strongly suggests that the proteins encoded by *Paracoccus* (pro)phages evolved via fusion of genes encoding the protease and major capsid protein. This is also in accordance with previous reports, e.g. regarding *Lactococcus* phage c2 structural proteins^36^. Such protein products were also predicted in the genomes of two of the active phages identified in this study: vB_PbeS_Pben1 (*pben1_p41*) and vB_PsuS_Psul1 (*psul1_p30*).

*Paracoccus* prophages encode endolysins which were classified as *N*-acetylmuramyol-L-alanine amidases (16 prophages), muramidases (18), peptidases M15 (15) and chitinases (4) (Supplementary Table S4). Interestingly, three prophages (vB_PsaS_PD29, vB_PsaS_PD48, and vB_PspS_PD38) also encode tail-associated peptidoglycan-degrading enzymes, that presumably facilitate phage DNA injection into the host cell in the initial stages of infection. Only in case of vB_PpaP_PD14 any protein resembled similarity to known endolysins was found.

It was also revealed that *Paracoccus* prophages encode an extensive repertoire of DNA modification proteins. In the genomes of 48 (out of 59) prophages, at least one DNA MTase gene was identified (Supplementary Table S4). Of the 88 predicted DNA MTases, 58 were classified as m^6^A or m^4^C DNA MTases and 30 as m^5^C DNA MTases. Although homologues of these MTases are abundant in sequence databases, their activity has yet to be confirmed by experimental data. Only in the case of Pd42_p05, encoded by vB_PspS_PD42, its specificity can be presumed (i.e. YGGCCR), based on its similarity (78% aa identity) to Pami1_p55 of vB_PamS_Pami1^11^.

**Figure 2:**
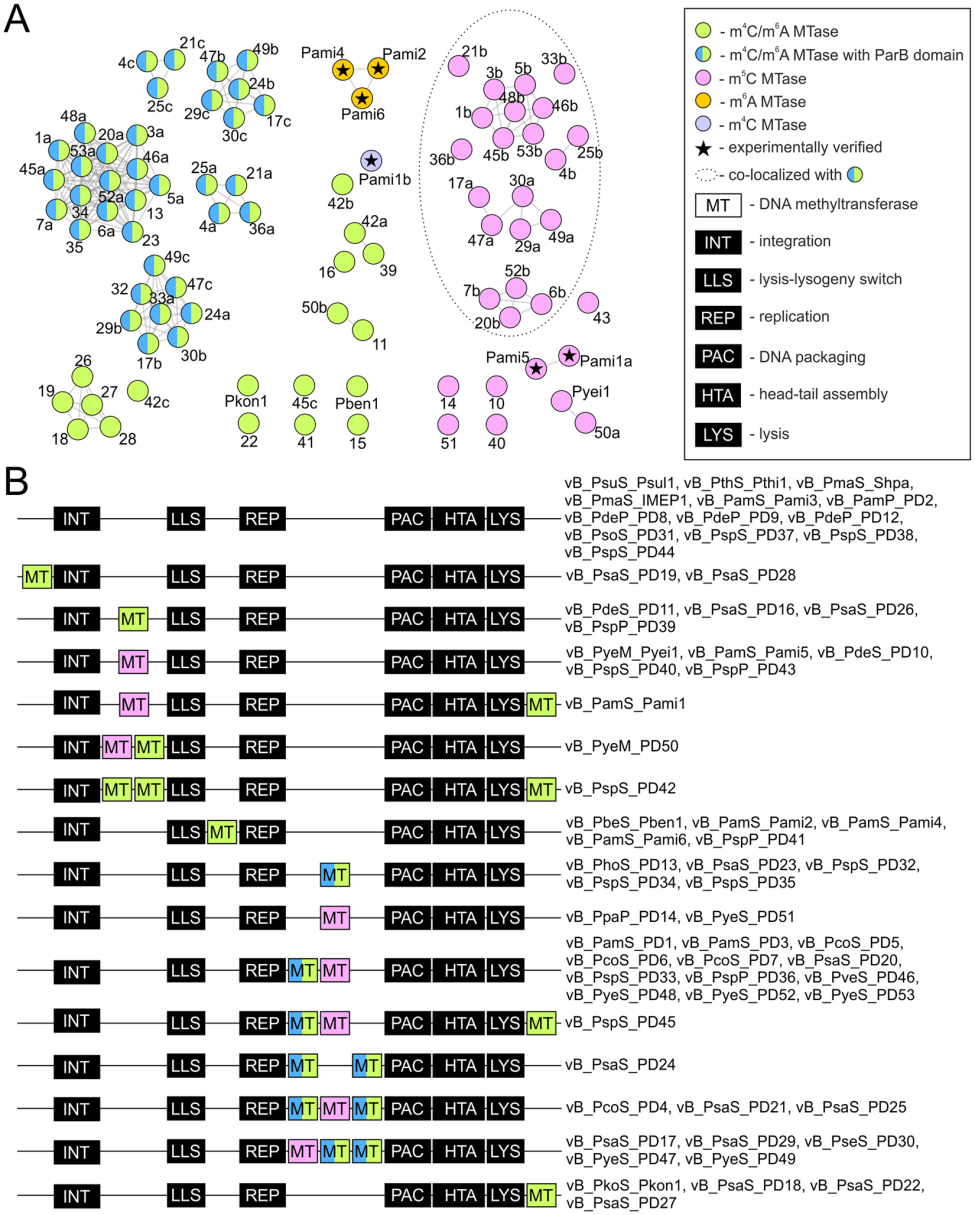
Diversity of DNA MTases of *Paracoccus* (pro)phages and genomic location of their genes. (**A**) General diversity of identified MTases as a network of MTases (nodes) connected with lines (edges) that reflect at least 80% amino acid sequence identity over at least 75% sequence coverage. The colours of the nodes representing single MTases, reflect the target base of their methylation, except the half blue-half green nodes, which additionally indicates the presence of a ParB domain at the N-terminus. Several nodes are also marked with stars to indicate experimental verification of their specificity. The labels/numbers give the names of the phage from which each MTase originates (e.g. Pami4 from vB_PamS_Pami4, 11 from vb_PdeS_PD11). This corresponds to data presented in Table 3. Where more than one MTase gene is present within a (pro)phage genome, the suffixes “a”, “b” or “c” are added to the label/number, corresponding to their order in that genome. (**B**) Simplified schematic representation of phage genomes showing virus-specific gene modules and the location of the MTase genes. MT blocks are coloured according to MTase target base specificity. The prophages that share each genome arrangement are listed on the right side of the genome diagrams.

The most numerous subgroup of DNA m^6^A/m^4^C MTases (36 examples) is comprised of enzymes containing the ParB domain within their N-terminal region (Fig. 2A). These genes were all found upstream of genes encoding the terminase subunits (PAC module) (Fig. 2B). The presence of MTase genes (and other DNA modifying genes) within a specified region of the phage genome (named the ParB-Tls locus), sandwiched between genes encoding a ParB-like protein and terminase large subunit has been reported previously^37^. It was suggested that phage-encoded ParB-like proteins may be involved in directing the DNA-modification apparatus to specific sites within the virus genome during packaging^37^. It was also shown that ParB proteins may be fused with the MTases^37^, as was observed in the case of the *Paracoccus* phages. Interestingly, in the predicted ParB-Tls loci of the *Paracoccus* phages, an additional 21 genes encoding m^5^C DNA MTases (lacking ParB domains) were found. Far fewer MTase genes were found at other locations, including downstream (12 genes) and upstream (2) of the integrase gene, or downstream of the lysis module (7) (Fig. 2B). Interestingly, MTase genes were located in the proximity of the replication modules in only four phages (including three related prophages of polylysogenic *P. aminophilus*), whereas such genomic localization of these genes was common in previously analyzed *Sinorhizobium* (also representatives of *Alphaproteobacteria*) prophages^11,31^.

Bacteriophages can also encode RM systems that may restrict the entry of other phage or plasmid DNA during lysogeny or reprogram gene expression^38^. In the *Paracoccus* prophage genomes, 13 RM systems (four type I, six type II and three type III) were identified (Supplementary Table S4).

As mentioned above, phages can carry auxiliary metabolic genes that may benefit their hosts. Interestingly, genes encoding proteins that potentially confer metal resistance were found in 10 phages. These are: (i, ii) tellurium resistance proteins TerB (phage vB_PamS_Pami1) and TerC (vB_PspS_PD44), (iii, iv) arsenite resistance protein ArsB (vB_PcoS_PD6 and vB_PsaS_PD20), (v, vi) zinc/cadmium/lead-transporting ATPase ZntA (vB_PhoS_PD13 and vB_PspS_PD34), (vii) multidrug efflux system AcrABCR (vB_PsaS_PD23), (viii) lead/cadmium/zinc/mercury transporter, copper transporting ATPase and a multi-copper oxidase (vB_PspS_PD33), (ix) cobalt transporter CorA (vB_PyeS_PD47) and (x) zinc transporter ZitB (vB_PyeS_PD49) (Supplementary Table S4). Heavy metal-rich regions are ubiquitous all over the planet and this is a consequence of natural processes (e.g. bioweathering of metal-containing minerals) and anthropogenic activities (e.g. burning of fossil fuels)^39^. *Paracoccus* spp. are frequently found in metal-rich environments (including metal mines and contaminated soils) and therefore acquisition of metal resistance genes may be beneficial for these bacteria^40–42^. Acquired (with a phage) resistance genes may modify bacterial host reaction to toxic elements and therefore enhance its overall fitness under detrimental, environmental conditions and, in a consequence, facilitates production of the virus progeny.

It was also shown that, in addition to two previously described phages, eight of *Paracoccus* prophages carried TA modules. The type of TA modules varied: two were RelBE-like, two HicAB-like, two VapBC-like, one ParDE-like and one HigBA-like (Supplementary Table S4).

### Comparative genomics of *Paracoccus* (pro)phages

In this study, 5 novel active lysogenic *Paracoccus* phages and 53 prophages were identified. These, together with six previously characterized prophages of *P. aminophilus* JCM 7686 (of which vB_PamS_Pami6 was shown to be an active lysogenic virus) and two lytic phages (vB_PmaS_IMPE1 and vB_PmaS_Shpa), constitute the current virome of the genus *Paraccocus*, which consists of 66 (pro)phages in total. This provided the opportunity for comprehensive genomic studies to reveal the common and unique features of these (pro)phages.

**Figure 3:**
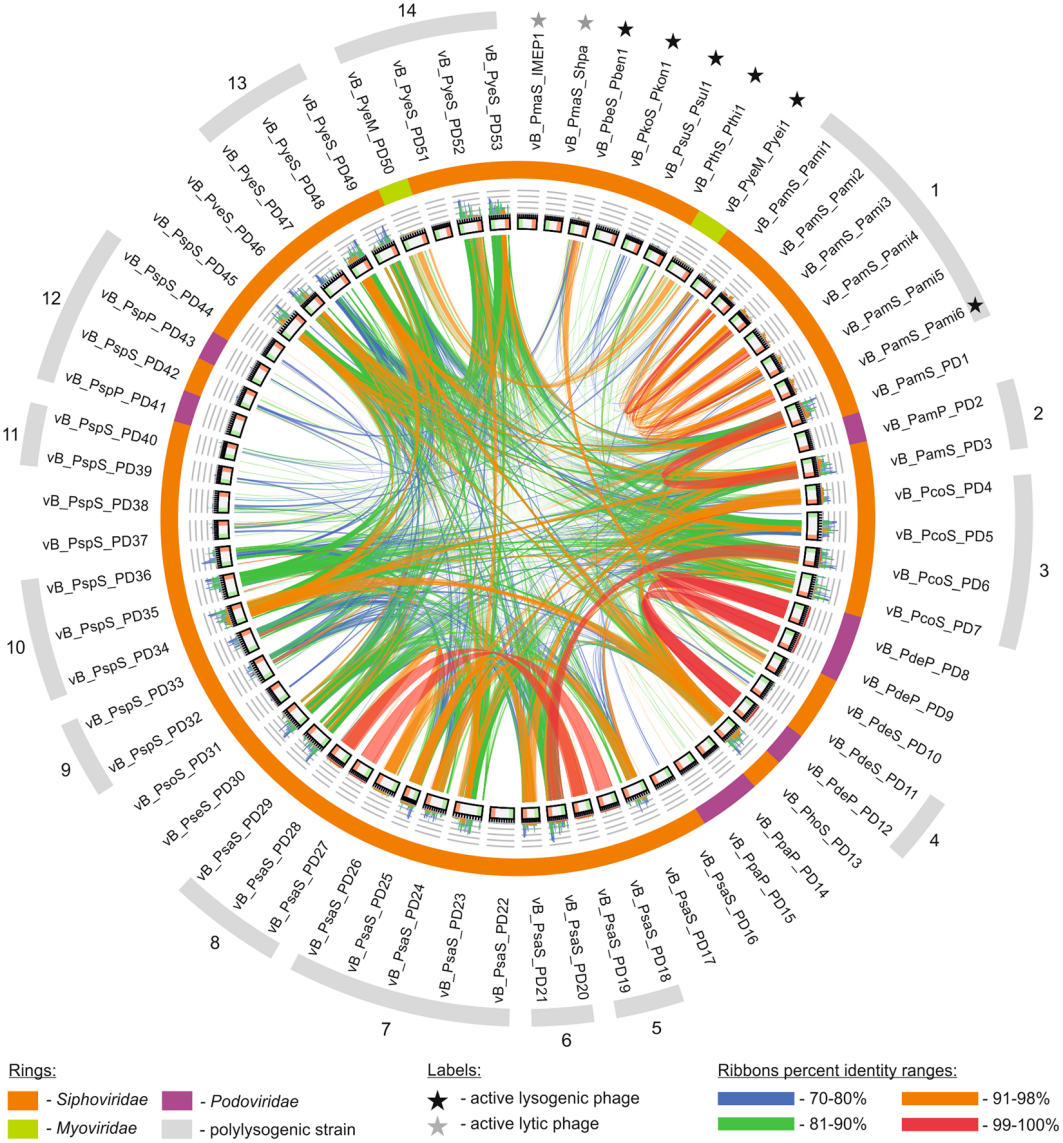
Comparison of *Paracoccus* (pro)phage genomes. Whole-genome similarity analysis was performed using Circoletto with e-100 as the threshold. The ribbon colours reflect the percentage identity of particular genomic regions. The bars within the first ring represent subsequent phages. The next ring, comprised of histograms, shows the frequency of hits in certain regions of the analyzed genomes. The outer-most ring reflects the (pro)phage classification: orange – *Siphoviridae*, green – *Myoviridae* and violet – *Podoviridae*. Outer-most, gray curves indicate polylysogenic host strains: 1 – *P. aminophilus* JCM 7686, 2 – *P. aminovorans* HPD-2, 3 – *P. contaminans* RKI, 4 - *P. denitrificans* PD1222, 5 – *P. sanguinis* 39524, 6 – *P. sanguinis* 4681, 7 – *P. sanguinis* 5503, 8 – *P. sanguinis* DSM 29303, 9 – *Paracoccus* sp. BM15, 10 – *Paracoccus* sp. CBA4604, 11 – *Paracoccus* sp. S4493, 12 – *Paracoccus* sp. SCN 68–21, 13 – *P. yeei* ATCC BAA-599, 14 – *P. yeei* TT13.

A first step in the comparison of *Paracoccus* (pro)phages was whole genome all-against-all BLASTn searches and their visualization with Circoletto (Fig. 3). This analysis revealed that at the nucleotide level (with a threshold e-value of <1e-100), 5 (pro)phages (lytic phages vB_PmaS_IMEP1 and vB_PmaS_Shpa (Shpa), and prophages vB_PsaS_PD22, vB_PspP_PD41 and vB_PyeS_PD51) were unique, 9 other prophages were nearly (if not) identical to at least one other, while the rest showed only local (limited to mostly short genomic regions) identities (of at least 73%).

The first group of identical prophages consists of members of the *Podoviridae*, vB_PdeP_PD8, vB_PdeP_PD9 and vB_PdeP_PD12, sharing 99–100% sequence identity, all of which were identified in closely related *P. denitrificans* strains. Another group is composed of two prophages, vB_PcoS_PD6 and vB_PsaS_PD20, sharing 99% sequence identity. Interestingly, both of these prophages carry an auxiliary gene encoding the arsenite efflux pump ArsB integrated in the opposite orientation to the surrounding structural genes. It is noteworthy that these prophages are present in strains isolated on different continents, from different environments and 10 years apart. Another two pairs of identical prophages are the Mu-like viruses vB_PsaS_PD18 and vB_PsaS_PD27, and vB_PsaS_PD19 and vB_PsaS_PD28, respectively. These were identified within the genomes of human blood-borne *P. sanguinis* strains 39524 and DSM 29303.

**Figure 4:**
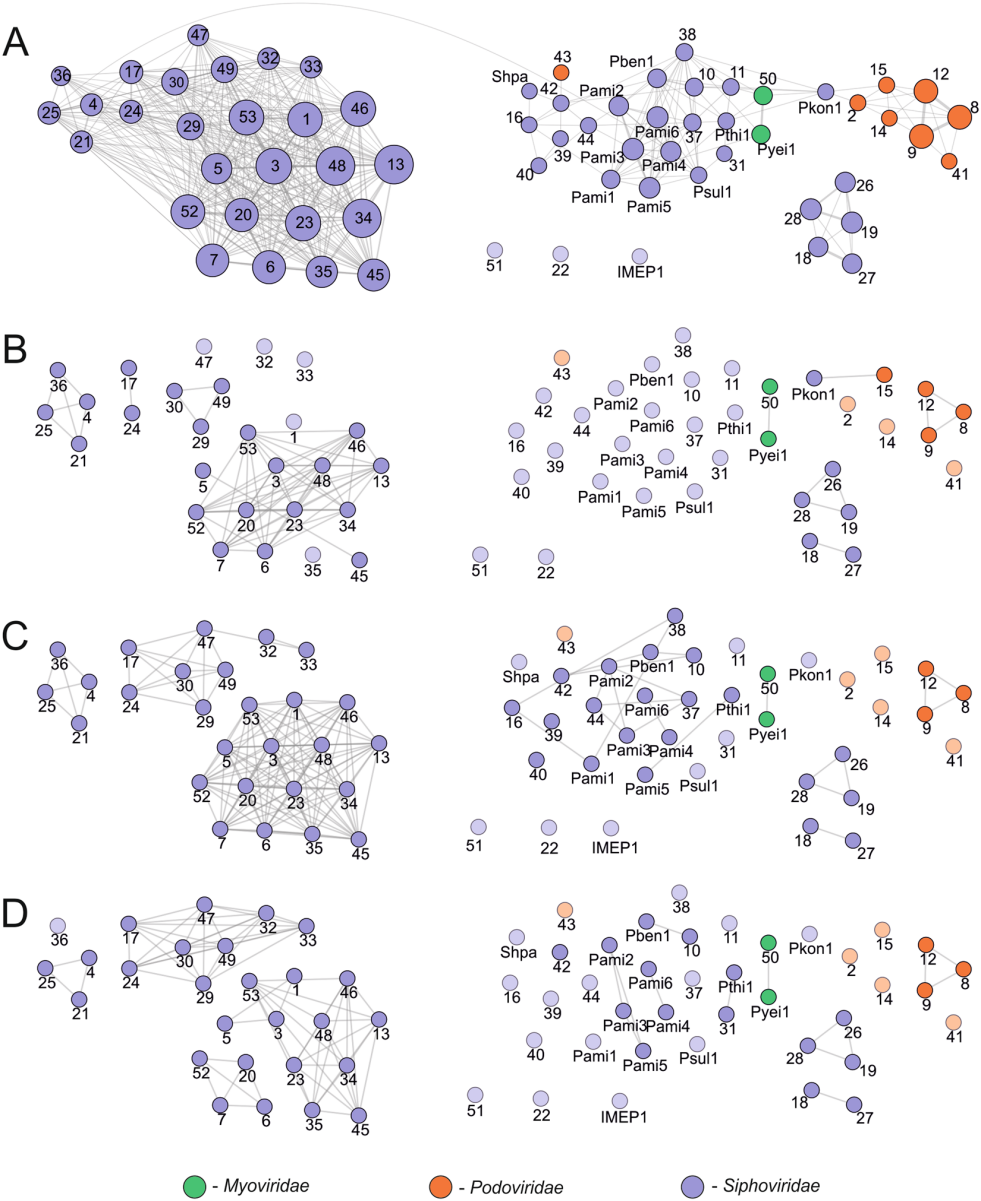
Protein-based similarity network of *Paracoccus* (pro)phages. The general clustering of (pro)phages based on their summarized proteomes (**A**), integrases (**B**), large terminase subunits (**C**) and major capsid proteins (**D**). Nodes represent a single (pro)phage, while edges correspond to the summarized quantity of reciprocally similar proteins. Orphan nodes are made transparent for better visibility. On (**A**) the size of the node corresponds to the number of prophages with which they share proteins. The nodes represent the following (pro)phages (*Paracoccus* strain): Pben1 – vB_PbeS_Pben1 (*P. bengalensis*); Pkon1 – vB_PkoS_Pkon1 (*P. kondratieve*); Psul1 – vB_PsuS_Psul1 (*P. sulfuroxidans*); Pthi1 – vB_PthS_Pthi1 (*P. thiocyanatus*); Pyei1 – vB_PyeM_Pyei1 (*P. yeei* CCUG 32053); IMEP1 – vB_PmaS_IMEP1 (*P. marcusii*); Shpa – vB_PmaS_Shpa (*P. marinus*); Pami1-Pami6 – vB_PamS_Pami1-vB_PamS_Pami6 (*P. aminophilus*); 1 – vB_PamS_PD1 (*P. aminovorans* DSM 8537); 2–3 – vB_PamP_PD2-vB_PamS_PD3 (*P. aminovorans* HPD-2); 4–7 – vB_PcoS_PD4-vB_PcoS_PD7 (*P. contaminans*); 8 – vB_PdeP_PD8 (*P. denitrificans* DSM 413); 9 – vB_PdeP_PD9 (*P. denitrificans* DSM 415); 10 – vB_PdeS_PD10 (*P. denitrificans* ISTOD1); 11–12 – vB_PdeS_PD11-vB_PdeP_PD12 (*P. denitrificans* PD1222); 13 – vB_PhoS_PD13 (*P. homiensis*); 14 – vB_PpaP_PD14 (*P. pantotrophus* DSM 1403); 15 – vB_PpaP_PD15 (*P. pantotrophus* J46); 16 – vB_PsaS_PD16 (*P. saliphilus*); 17 – vB_PsaS_PD17 (*P. sanguinis* 10990); 18–19 – vB_PsaS_PD18-vB_PsaS_PD19 (*P. sanguinis* 39524); 20–21 – vB_PsaS_PD20-vB_PsaS_PD21 (*P. sanguinis* 4681); 22–26 – vB_PsaS_PD22-vB_PsaS_PD26 (*P. sanguinis* 5503); 27–29 – vB_PsaS_PD27-vB_PsaS_PD29 (*P. sanguinis* DSM 29303); 30 – vB_PseS_PD30 (*P. sediminis*); 31 – vB_PsoS_PD31 (*P. solventivorans*); 32–33 – vB_PspS_PD32-vB_PspS_PD33 (*Paracoccus* sp. BM15); 34–36 – vB_PspM_PD34-vB_PspS_PD36 (*Paracoccus* sp. CBA4604); 37 – vB_PspS_PD37 (*Paracoccus* sp. J39); 38 – vB_PspS_PD38 (*Paracoccus* sp. N5); 39–40 – vB_PspS_PD39-vB_PspS_PD40 (*Paracoccus* sp. S4493); 41–44 – vB_PspP_PD41-vB_PspS_PD44 (*Paracoccus* sp. SCN 68–21); 45 – vB_PspS_PD45 (*P. sphaerophysae*); 46 – vB_PveS_PD46 (*P. versutus*); 47–49 – vB_PyeS_PD47-vB_PyeS_PD49 (*P. yeei* ATCC BAA-599); 50–53 – vB_PyeM_PD50-vB_PyeS_PD53 (*P. yeei* TT13).

Comparison of the *Paracoccus* (pro)phages was continued by constructing protein-based similarity networks (Fig. 4A). In total, the 66 analyzed (pro)phages encode 3,891 putative proteins, of which 2,062 are similar to at least one other protein. Comparative analysis of whole proteomes showed that the *Paracoccus* (pro)phages can be grouped into three major clusters and three orphan nodes (representing: vB_PmaS_IMEP1, vB_PsaS_PD22 and vB_PyeS_PD53). The densest (i.e. the most similar to one another) cluster is composed of a set of viruses of the *Siphoviridae*, while the most numerous cluster (31 elements) is more relaxed, reflecting a lower number of reciprocally similar proteins of phages that comprise this group (Fig. 4A). It is important to mention that these clusters are linked via a common protein – a truncated IS*3* family transposase encoded by vB_PsaS_PD25 and vB_PamS_Pami4 (Fig. 4A). The relaxed cluster contains *Podoviridae* (pro)phages on the peripheries, while the core is built by representatives of *Siphoviridae* and *Myoviridae*. Interestingly, vB_PkoS_Pkon1 constitutes an internal linker within this cluster because it encodes proteins (e.g. endolysin Pkon1_p73 exhibiting similarity to appropriate proteins of vB_PdeS_PD10, vB_PdeS_PD11 and vB_PthS_Pthi1) whose homologues are present in proteomes of *Paracoccus* phages classified to the *Siphoviridae* and *Podoviridae*. The third cluster of similar phages consists of all five Mu-like *Siphoviridae* viruses identified within the opportunistic human pathogens *P. sanguinis* 39542 and DSM 29303.

Several polylysogenic host strains were identified in this study and we checked the reciprocal similarity of their phage proteomes. Within *P. aminophilus* JCM 7686 and *Paracoccus* sp. BM15, all identified prophages (six and two, respectively) show similarity of their encoded proteins; they share between five and 30 highly similar proteins. It is also worth mentioning that *P. aminophilus* JCM 7686 contains the highest number of prophages^11^. Some prophages of the other polylysogenic host strains also exhibit similarities, including (i) three (out of four) prophages of *P. contaminans* RKI (vB_PcoS_PD5-PD7) that share between 14 and 29 common proteins, (ii) two (out of three) prophages of *Paracoccus* sp. CBA4604 (vB_PspS_PD34 and vB_PspS_PD35), sharing 19 proteins, (iii) two (out of three) prophages of *P. yeei* ATCC BAA-599 (vB_PyeS_PD47 and vB_PyeS_PD49), sharing 11 proteins and (iv) two (out of four) prophages of *P. yeei* TT13 (vB_PyeS_PD52 and vB_PyeS_PD53), sharing 16 proteins. In contrast, of the five prophages of *P. sanguinis* 5503, only vB_PsaS_PD23 shares a single protein with vB_PsaS_PD24 and vB_PsaS_PD25. Similarly, amongst four prophages of *Paracoccus* sp. SCN 68-21 only vB_PspS_42 and vB_PspS_43 encode a single similar protein.

Detailed analysis of the (pro)phage integration module sequences showed that they group into 10 clusters and 30 unique nodes (Fig. 4B). The largest cluster is composed of 11 (out of 15) serine recombinases for which integration sites were not identified. The four remaining serine recombinases (of vB_PamS_Pami6, vB_PamS_PD1, vB_PsoS_PD31 and vB_PsaS_PD35) form single nodes. It is worth emphasizing that the specific integration site was predicted (tRNA_Met_ gene^11^) for only one phage encoding serine recombinase – the active lysogenic phage vB_PamS_Pami6 (Supplementary Fig. S1). This analysis revealed that 10 other (pro)phages also integrated within the tRNA_Met_ gene, but these viruses all encode tyrosine recombinases (Supplementary Fig. S1). Another group is composed of *Podoviridae* (pro)phages (i.e. vB_PdeP_PD8, vB_PdeP_PD9, vB_PdeP_DP12, vB_PamP_PD2, vB_PspP_PD41), integrated into tRNA_Thr_ genes with various anticodons (Supplementary Fig. S1). Interestingly, one podovirus, vB_PpaP_PD15, encodes a tyrosine recombinase highly similar (92.7%) to that of siphovirus vB_PkoS_Pkon1 and both have integrated into OmpR family transcriptional regulator genes. Another two clusters group phages encoding Mu-like transposases (Supplementary Fig. S1).

The other two networks of large terminase subunits and major capsid protein sequences show a higher level of conservation among these proteins than in the case of recombinases (Fig. 4C,D). In both cases, 12 clusters grouping between two and 15 (pro)phages were found. Moreover, there is significant congruency between these networks, which is especially visible amongst Mu-like prophages, myoviruses and podoviruses (Fig. 4C,D). This finding, together with our previous observations for *Sinorhizobium* (pro)phages, indicates that terminase large subunits and major capsid proteins as markers representing congruent clustering are the most convenient tools for phylogenetic analyses of alphaproteobacterial viruses^31^.

#### Diversity of Paracoccus (pro)phages in comparison to the general diversity of bacteriophages

**Figure 5:**
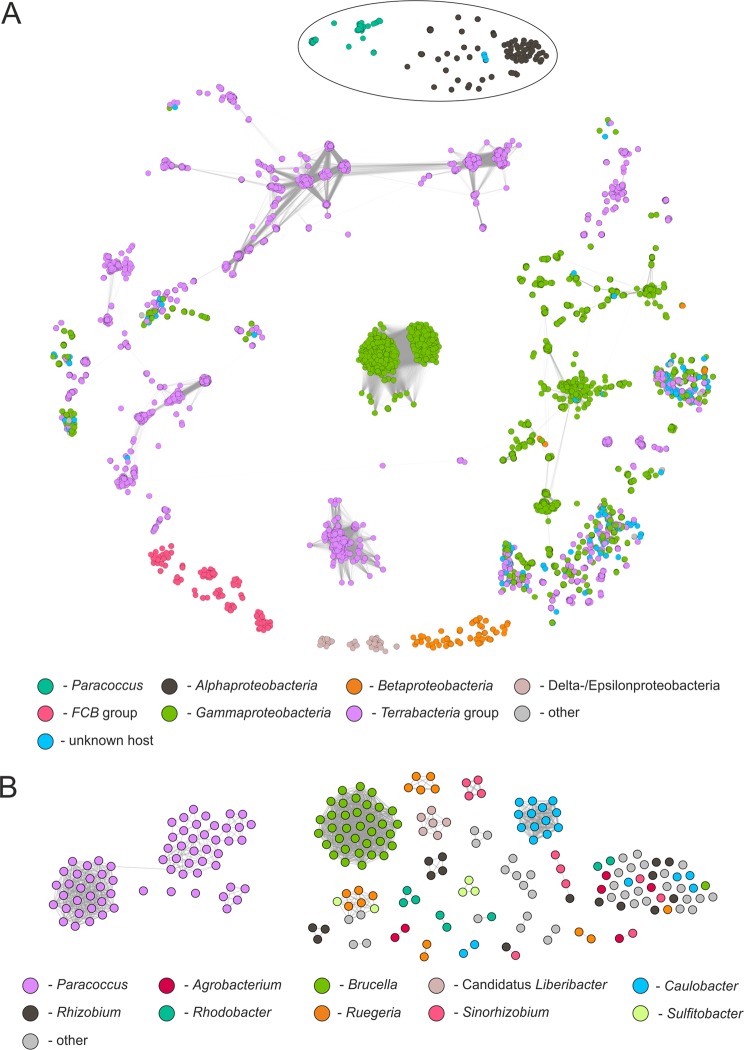
Protein-based similarity network analysis of *Paracoccus* (pro)phages and other bacteriophages retrieved from the NCBI Viruses database. (**A**) Overall similarity network of known bacterial phages. Nodes are coloured based on the taxonomy of the phage host (at phylum level, except *Proteobacteria* where classes are considered). *Paracoccus* (pro)phages are distinguished within the network. The host taxonomy is based on manually-curated qualifiers in the source section and organism name of the virus GenBank files. (**B**) Magnified image of *Alphaproteobacteria* (pro)phage network. The colour scheme is based on the host genus classification of the phages. The topology of the clustering of *Paracoccus* phages is the same as the one presented on Fig. 4, where (pro)phages were coloured based on their classification to *Sipho*-, *Myo*- and *Podoviridae* families.

*Paracoccus* (pro)phages were subjected to comparative analyses with all bacteriophage genomes deposited in the NCBI Viruses database by constructing a complex protein similarity network composed of 6,126 nodes and 330,592 edges (Fig. 5). Interestingly, *Paracoccus* phages created separate clusters (Fig. 5).

We focused on detailed analysis of the relationship between phages infecting bacteria of the *Alphaproteobacteria* class. In the analyzed network, 156 *Alphaproteobacteria* phages, infecting hosts from 38 different genera were included. Our analysis revealed that these viruses are highly diverse and do not show similarity to other phages (Fig. 5A). They created 24 clusters (between two and 35 nodes) and 40 orphan nodes. The two most numerous clusters are composed of 35 *Brucella* phages and 13 *Caulobacter* phages. Interestingly, there are only five multi-host clusters grouping phages that infect *Alphaproteobacteria* representing various genera. The largest of such clusters groups nine related phages infecting *Reugeria* (five phages), *Dinoroseobacter* (two) and *Sulfitobacter* (two) (Fig. 5B).

The network analysis (Fig. 5) showed the distinction of *Alphaproteobacteria* phages from other bacteriophages and hence, searching for potential links between these phages and other viruses, we have used the IMG/VR database resources^43^. Amongst over 700,000 viral contigs present within the IMG/VR database, less than 1% (4,915) encoded at least a single protein similar to those of *Alphaproteobacteria* phages. From these viral contigs only 212 with completeness parameter over 75% were overlaid onto the global network (Supplementary Fig. S2). As a result, it was shown, that vB_PkoS_Pkon1 has been connected with *Gammaproteobacteria* phages (via pkon1_p50, hypothetical protein with the P63C domain), while vB_PmaS_Shpa, vB_PsaS_PD19, vB_PsaS_PD26 and vB_PsaS_PD28 have been linked with other *Alphaproteobacteria* phages (via single-stranded DNA-binding protein and large terminase subunit protein). Interestingly, newly added contigs retrieved from the IMG/VR database extended many other clusters and linked *Alphaprotoebacteria* phages with viruses infecting *Terrabacteria* and *Gammaproteobacteria* (Supplementary Fig. S2). However, despite several new links/connections, this extended network analysis still indicates that *Alphaproteobacteria* (and particularly *Paracoccus*) phages create separate groups.

It is important to mention that since the number of alphaproteobacterial virus genomes currently available for comparison is low (especially compared to the number of other phage genomes in the NCBI database), all performed analyses still have some limitations. Therefore, network analyses should be repeated once the database has been enriched in the future.

## Conclusion

In this study, five novel active temperate phages and 53 prophages of *Paracoccus* spp. (*Alphaproteobacteria*) were identified and analyzed together with six previously identified prophages of *P. aminophilus* JCM 7686 and two lytic phages (vB_PmaS_IMEP1 and vB_PmaS_Shpa). Four of the newly discovered active phages represent the *Siphoviridae* family, while vB_PyeM_Pyei1 is the first active *Myoviridae* phage infecting *Paracoccus* spp. Moreover, amongst the identified prophages, the first *Podoviridae* viruses infecting *Paracoccus* spp. were distinguished. Several auxiliary metabolic genes were found within the genomes of the identified *Paracoccus* (pro)phages. These genes encode proteins that potentially confer metal resistance. This may be highly beneficial to bacterial hosts, as many *Paracoccus* spp. have been isolated from various contaminated environments. Amongst other genes found within analysed (pro)phages, these encoding DNA methyltransferases are very common. It was shown that, 58 of identified methylases were classified as m^6^A/m^4^C DNA MTases and 30 as m^5^C DNA MTases. In similarity network analysis, these MTases formed highly conserved clusters, possibly grouping enzymes with common specificities. Interestingly, 57 genes encoding MTases were localized in a common region, i.e. the ParB-Tls locus. This location was also shared by a large group of genes encoding MTases fused with ParB-like proteins, that may be involved in directing the DNA-modification apparatus during packaging. Finally, it was shown that *Paracoccus* (pro)phages form a separate group of viruses, that is not only distinct from other phages of *Alphaproteobacteria*, but also from all other bacterial viruses.

## Methods

### Bacterial strains, plasmids and culture conditions

The following strains were used in this study: *E. coli* DH5α^44^, *P. alcaliphilus* JCM 7364^45^, *P. aminophilus* JCM 7686^3^, *P. aminovorans* JCM 7685^3^, *P. alkenifer* DSM 11593^2^, *P. bengalensis* DSM 17099^4^, *P. ferroxidans* NCCB 1300066^46^, *P. haeundaensis* LGM P-21903^5^, *P. halophilus* JCM 14014T^6^, *P. homiensis* DSM 17862^47^, *P. kondratievae* NCIMB 13773T^48^, *P. pantotrophus* DSM 11072^49^, *P. seriniphilus* DSM 14827^7^, *P. solventivorans* DSM 11592^2^, *P. sulfuroxidans* JCM 14013^50^, *P. thiocyanatus* JCM 20756^51^, *P. versutus* UW1R^52^ and UW225 (Rif^r^-derivative of a wild-type strain)^53^, and *P. yeei* CCUG 32053^54^. All strains were grown in lysogeny broth (LB) medium at 37 °C (*E. coli*) and 30 °C (*Paracoccus* spp.). Liquid cultures were incubated with shaking. When required, media were supplemented with kanamycin (50 μg ml^−1^) and rifampin (50 μg ml^−1^). Plasmid pABW3 was used for the stability testing^52^.

### Standard molecular biology procedures

Standard DNA manipulation methods were performed as described by Sambrook and Russell (2001)^55^. Transformation of *E. coli* strains and triparental mating of *P. versutus* were performed according to previously described methods^52,56^. The test for the presence of cohesive ends of the phage genome was performed as previously described^57^, using various restriction enzymes (Thermo Fisher Scientific, Waltham, MA, USA).

### Cloning of the toxin-antitoxin systems

Toxin-antitoxin (TA) systems of vB_PbeS_Pben1 and vB_PkoS_Pkon1 were PCR amplified using Phusion High-Fidelity DNA Polymerase (Thermo Fisher Scientific) with appropriate primer pairs, i.e. TABamHf 5′-GTTGTTGGATCCATGATCTCGGCATCAGCAG-3′ and TAEcoRr 5′-GGTGGTGAATTCAACACATTGCAGCAATGCTC-3′ for the TA module of vB_PbeS_Pben1, and PKTABamHI 5′-TGCAGGATCCAATACCGCATCCGTTCG-3′ and PKTAEcoRI 5′-AGCTGAATTCCATGGCCGCCTCAATCC-3′ for the TA module of vB_PkoS_Pkon1 (introduced restriction sites are underlined). The following PCR program was applied using a Mastercycler (Eppendorf, Hamburg, Germany) to amplify the desired products: initial denaturation at 95 °C for 3 min followed by 35 cycles of denaturation at 98 °C for 20 s, annealing at 64 °C for 1 min, extension at 72 °C for 1 min/kb and then a final extension at 72 °C for 1 min/kb. The obtained PCR amplicons were analyzed by agarose gel electrophoresis and purified using a Gel Out kit (A&A Biotechnology, Gdynia, Poland). The DNA fragments were then digested with EcoRI and BamHI and cloned in the vector pABW3 cleaved with the same restriction endonucleases. The resulting plasmid constructs were named pABW3-TA_PBE and pABW3-TA_PKO, respectively.

### Mitomycin induction of prophages and purification of phage particles for DNA preparation and transmission electron microscopy

*Paracoccus* cultures were grown to an OD_600_ of 0.4, then mitomycin C (Sigma-Aldrich, St. Louis, MO, United States) was added to 500 µg ml^−1^ and incubation was continued for 6 h. The cells were then pelleted by centrifugation and phage particles in the supernatant precipitated using PEG/NaCl^55^. Bacteriophage particles were collected by centrifugation and resuspended in SM buffer^55^. Phage DNA was isolated by phenol-chloroform extraction and isopropanol precipitation^55^, and analyzed by 0.7% agarose gel electrophoresis. For transmission electron microscopy (TEM) analysis, phage particles were purified on a 1 ml Convective Interaction Media (CIM^®^) anion-exchange monolith column (BIA Separations, Ajdovscina, Slovenia) using an ÄKTApurifier system (GE Healthcare, Little Chalfont, UK) running UNICORN™ software, according to a recently published protocol^58^. Briefly, phages from the initial purification were loaded on the column at a flow rate of 2 ml/min. Impurities were then washed out by increasing the proportion of elution buffer (20 mM Tris-HCl, pH 7.5; 2 M NaCl) in the mobile phase from 0 to 10%, at a flow rate of 4 ml/min. Elution of the phage particles was achieved by increasing the proportion of elution buffer to 35%.

### Transmission electron microscopy (TEM)

For TEM analysis, 10 μl samples of purified phage were adsorbed onto carbon-coated grids (Sigma-Aldrich) for 3 min, stained with 1.5% uranyl acetate (Sigma-Aldrich) and examined using a Tecnai Spirit BioTWIN transmission electron microscope (FEI Company, Hillsboro, OR, USA). Images were collected using iTEM software (FEI Company). The visualization of phages was performed at the Laboratory of Electron Microscopy, Faculty of Biology, University of Gdansk, Gdansk, Poland.

### Determination of phage host range by spot testing

To determine bacterial susceptibility to phage-mediated lysis, 17 *Paracoccus* strains (*P. alcaliphilus* JCM 7364, *P. aminophilus* JCM 7686, *P. aminovorans* JCM 7685, *P. alkenifer* DSM 11593, *P. bengalensis* DSM 17099, *P. ferroxidans* NCCB 1300066, *P. haeudaensis* LGM P-21903, *P. halophilus* JCM 14014T, *P. homiensis* DSM 17862, *P. kondratievae* NCIMB 13773T, *P. pantotrophus* DSM 11072, *P. seriniphilus* DSM 14827, *P. solventivorans* DSM 11592, *P. sulfuroxidans* JCM 14013, *P. thiocyanatus* JCM 20756, *P. versutus* UW1R and *P. yeei* CCUG 32053) were grown in liquid LB medium and plated onto LB agar plates. A drop of each phage suspension was spotted onto the bacterial lawns and the plates were incubated at 30 °C. The plates were examined for evidence of bacterial lysis after 72 h.

### Plasmid stability testing

The stability of plasmids in *P. versutus* cells was tested as described previously^25^. Briefly, *P. versutus* UW225 containing the introduced plasmids (pABW3-TA_PKO, pABW3-TA_PBE or pABW3 as a control) were grown overnight at 30 °C in LB medium supplemented with kanamycin. Stationary phase cultures were then diluted in fresh medium without added antibiotic and cultivated for approximately 10, 20 or 30 generations. Samples were diluted and plated onto solid medium lacking kanamycin. One hundred colonies from each plate were then tested for the presence of the selection marker by replica plating. The percentage of kanamycin resistant colonies was used as a measure of the retention of the different plasmids. All plasmid stability assays were performed in triplicate.

### DNA sequencing and phage genome assembly

The complete nucleotide sequences of *Paracoccus* phages were determined in the DNA Sequencing and Oligonucleotide Synthesis Laboratory (oligo.pl) at the Institute of Biochemistry and Biophysics, Polish Academy of Sciences. The phage genomes were sequenced using an Illumina MiSeq instrument in paired-end mode with a v3 chemistry kit. The obtained sequence reads were filtered for quality with cutAdapt v1.15 trimming bases on 3′ ends (with quality lower than Q20) and removing reads containing Ns or shorter than 50 bp^59^. Processed reads were afterwards assembled using Newbler v3.0 software (Roche, Basel, Switzerland) with default settings. Final gap closure was performed by capillary sequencing of PCR amplicons using an ABI3730xl DNA Analyser (Applied Biosystems, Waltham, MA, USA).

### Prophage detection and classification

On June 25^th^ 2018, 9 complete and 55 draft *Paracoccus* species genomes were retrieved from the National Center for Biotechnology Information (NCBI) genome browser. The sequences of these genomes together with all complete plasmids of *Paracoccus* spp., were screened for the presence of prophages using PhiSpy^33^ and the results were verified by manual inspection. Assessment of the prophage genome completeness was based on the presence of modules responsible for phage integration, lysis/lysogeny switch, DNA packaging, head-tail assembly and lysis.

Taxonomy assignment of all prophages was conducted using the VIRFAM service, which also allowed more precise identification of certain structural proteins^34^.

### Genome annotation

The identified prophage sequences, as well as those of the mitomycin C-induced *Paracoccus* phages, were manually annotated using Clone Manager (Sci-Ed8) and Artemis software^60^. Annotation was based on homology searches performed using BLAST programs, including domain searches with CD-Search^61^. Putative tRNA genes were identified with the tRNAScan-SE 2.0 and ARAGORN programs^62,63^. Methyltransferase classification was performed using the REBASE database^64^ and manual inspection. For the identification of transmembrane proteins, i.e. holins, TMHMM^65^ and TMPRED (https://embnet.vital-it.ch/software/TMPRED_form.html) were used. The annotation of the identified heavy metal resistance genes was assisted by searches against the BacMet and PRIAM databases^66,67^.

### Comparative genomics

Phage genome comparisons were performed with the Circoletto tool, using an e-value of 1e-100 as the threshold^68^. The construction of similarity networks was based on all-against-all BLASTp comparisons of three sets of proteomes: (i) those derived from 66 *Paracoccus* (pro)phages, (ii) those of the *Paracoccus* (pro)phages combined with all 6,253 viruses infecting *Bacteria* available in the NCBI genome browser (as of August 3^rd^ 2018), and (iii) the previous two datasets extended with the part of the IMG/VR database version 3 (as of July 1^st^ 2018)^43^. From the set of bacteriophages deposited in the NCBI database, 191 not encoding any proteins based on their annotations were excluded from the analyses. For the construction of the network only the IMG/VR viral contigs encoding at least a single protein similar to proteins encoded by known *Alphaproteobacteria* phages were used. From 4,915 resulting viral contigs (out of 760,453), these duplicating the nodes and with parameter of the genome completeness below 75% were excluded from further analysis. The following thresholds were used during the BLASTp searches: e-value 1e-10 (to avoid losing small, i.e. <100 aa, proteins from the analysis), query coverage of HSP of at least 75% and sequence identity of 80%. Within the obtained networks, each node represents a single (pro)phage (or IMG/VR-retrieved viral contig) and each edge corresponds to a common reciprocated similarity of at least one protein encoded by two connected (pro)phages or viral contigs. The thickness of the edge reflects the number of common proteins between two analyzed (pro)phages. These networks were created using self-written Python scripts and visualized in Gephi^69^ using ForceAtlas 2 layout^70^.

### Nucleotide sequence accession numbers

The nucleotide sequences of the vB_PbeS_Pben1, vB_PkoS_Pkon1, vB_PsuS_Psul1, vB_PthS_Pthi1 and vBPyeM_Pyei1 phages have been deposited in the GenBank (NCBI) database with the accession numbers MK291441, MK291442, MK291443, MK291444 and MK291445, respectively.

## Supplementary information

Supplementary Information 1

Supplementary Dataset 1

## Data Availability

All data generated or analyzed during this study are included in the manuscript and the Supplementary Information 1 and Supplementary Dataset 1. The nucleotide sequences of identified phages have been deposited in the GenBank (NCBI) database.
