# Supplementary material for: Characterization of the virome of Paracoccus spp. (Alphaproteobacteria) by combined in silico and in vivo approaches
Source: Sci Rep. 2019 May 27;9:7899. doi: 10.1038/s41598-019-44460-4 (PMC6536676; doi:10.1038/s41598-019-44460-4)
Supplement: Supplementary file 1 — Supplementary Information 1 [file 41598_2019_44460_MOESM1_ESM.pdf]

Characterization of the virome of *Paracoccus* spp. (*Alphaproteobacteria*) by combined *in silico* and *in vivo* approaches

(Supplementary Information 1)

Przemyslaw Decewicz[1], Lukasz Dziewit[1]*, Piotr Golec[1], Patrycja Kozlowska[2], Dariusz Bartosik[1], Monika Radlinska[2]

[1] University of Warsaw, Faculty of Biology, Institute of Microbiology, Department of Bacterial Genetics, Miecznikowa 1, 02-096 Warsaw, Poland
[2] University of Warsaw, Faculty of Biology, Institute of Microbiology, Department of Virology, Miecznikowa 1, 02-096 Warsaw, Poland

*** Corresponding author:** Lukasz Dziewit
University of Warsaw, Faculty of Biology,
Institute of Microbiology,
Department of Bacterial Genetics,
Miecznikowa 1, 02-096 Warsaw, Poland
tel: 48 225541406; fax: 48 225541402;
e-mail: ldziewit@biol.uw.edu.pl

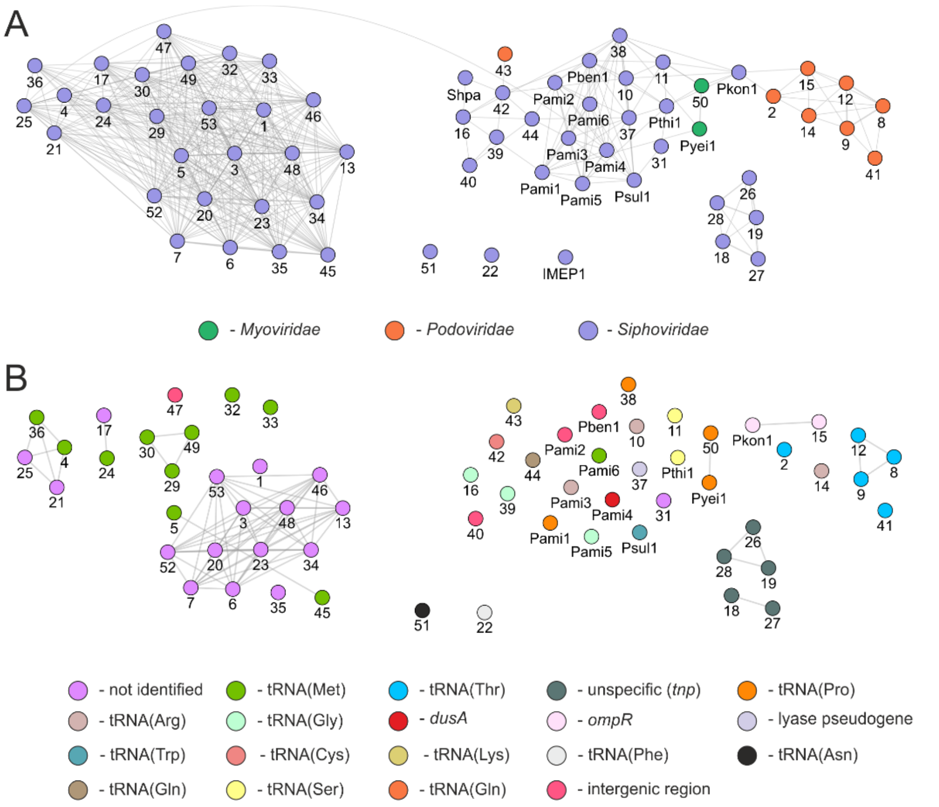

Node label	Phage name	Host	Node label	Phage name	Host
Pben1	vB PbeS Pben1	*P. bengalensis* DSM 17099	21	vB PsaS PD21	*P. sanguinis* 4681
Pkon1	vB PkoS Pkon1	*P. kondratieve* NCINM 13773	22	vB PsaS PD22	*P. sanguinis* 5503
Psul1	vB PsuS Psul1	*P. sulfuroxidans* JCM 14013	23	vB PsaS PD23	*P. sanguinis* 5503
Pthi1	vB PthS Pthi1	*P. thiocyanatus* JCM 20756	24	vB PsaS PD24	*P. sanguinis* 5503
Pyei1	vB PyeM Pyei1	*P. yeei* CCUG32053	25	vB PsaS PD25	*P. sanguinis* 5503
IMEP1	vB PmaS IMEP1	*P. marcusii*	26	vB PsaS PD26	*P. sanguinis* 5503
Shpa	vB PmaS Shpa	*P. marinus*	27	vB PsaS PD27	*P. sanguinis* DSM 29303
Pami1	vB PamS Pami1	*P. aminophilus* JCM 7686	28	vB PsaS PD28	*P. sanguinis* DSM 29303
Pami2	vB PamS Pami2	*P. aminophilus* JCM 7686	29	vB PsaS PD29	*P. sanguinis* DSM 29303
Pami3	vB PamS Pami3	*P. aminophilus* JCM 7686	30	vB PseS PD30	*P. sediminis* DSM 26170
Pami4	vB PamS Pami4	*P. aminophilus* JCM 7686	31	vB PsoS PD31	*P. solventivorans* DSM 6637
Pami5	vB PamS Pami5	*P. aminophilus* JCM 7686	32	vB PspS PD32	*Paracoccus* sp. BM15
Pami6	vB PamS Pami6	*P. aminophilus* JCM 7686	33	vB PspS PD33	*Paracoccus* sp. BM15
1	vB PamS PD1	*P. aminovorans* DSM 8537	34	vB PspM PD34	*Paracoccus* sp. CBA4604
2	vB PamP PD2	*P. aminovorans* HPD-2	35	vB PspS PD35	*Paracoccus* sp. CBA4604
3	vB PamS PD3	*P. aminovorans* HPD-2	36	vB PspS PD36	*Paracoccus* sp. CBA4604
4	vB PcoS PD4	*P. contaminans* RKI	37	vB PspS PD37	*Paracoccus* sp. J39
5	vB PcoS PD5	*P. contaminans* RKI	38	vB PspS PD38	*Paracoccus* sp. N5
6	vB PcoS PD6	*P. contaminans* RKI	39	vB PspS PD39	*Paracoccus* sp. S4493
7	vB PcoS PD7	*P. contaminans* RKI	40	vB PspS PD40	*Paracoccus* sp. S4493
8	vB PdeP PD8	*P. denitrificans* DSM 413	41	vB PspP PD41	*Paracoccus* sp. SCN 68-21
9	vB PdeP PD9	*P. denitrificans* DSM 415	42	vB PspS PD42	*Paracoccus* sp. SCN 68-21
10	vB PdeS PD10	*P. denitrificans* ISTOD1	43	vB PspP PD43	*Paracoccus* sp. SCN 68-21
11	vB PdeS PD11	*P. denitrificans* PD1222	44	vB PspS PD44	*Paracoccus* sp. SCN 68-21
12	vB PdeP PD12	*P. denitrificans* PD1222	45	vB PspS PD45	*P. sphaerophysae* HAMBI 3106
13	vB PhoS PD13	*P. homiensis* DSM 17862	46	vB PveS PD46	*P. versutus* DSM 582
14	vB PpaP PD14	*P. pantotrophus* DSM 1403	47	vB PyeS PD47	*P. yeei* ATCC BAA-599
15	vB PpaP PD15	*P. pantotrophus* J46	48	vB PyeS PD48	*P. yeei* ATCC BAA-599
16	vB PsaS PD16	*P. saliphilus* DSM 18447	49	vB PyeS PD49	*P. yeei* ATCC BAA-599
17	vB PsaS PD17	*P. sanguinis* 10990	50	vB PyeM PD50	*P. yeei* TT13
18	vB PsaS PD18	*P. sanguinis* 39524	51	vB PyeS PD51	*P. yeei* TT13
19	vB PsaS PD19	*P. sanguinis* 39524	52	vB PyeS PD52	*P. yeei* TT13
20	vB PsaS PD20	*P. sanguinis* 4681	53	vB PyeS PD53	*P. yeei* TT13

Figure S1. The variety of integration sites of *Paracoccus* (pro)phages. On the upper panel (A), clustering based on (pro)phage proteome is presented, while on the lower panel (B) the edges correspond to the similarity between the integrases (1e-10, at least 80% sequence identity, at least 75% query coverage) and nodes are coloured according to the recognized integration site.

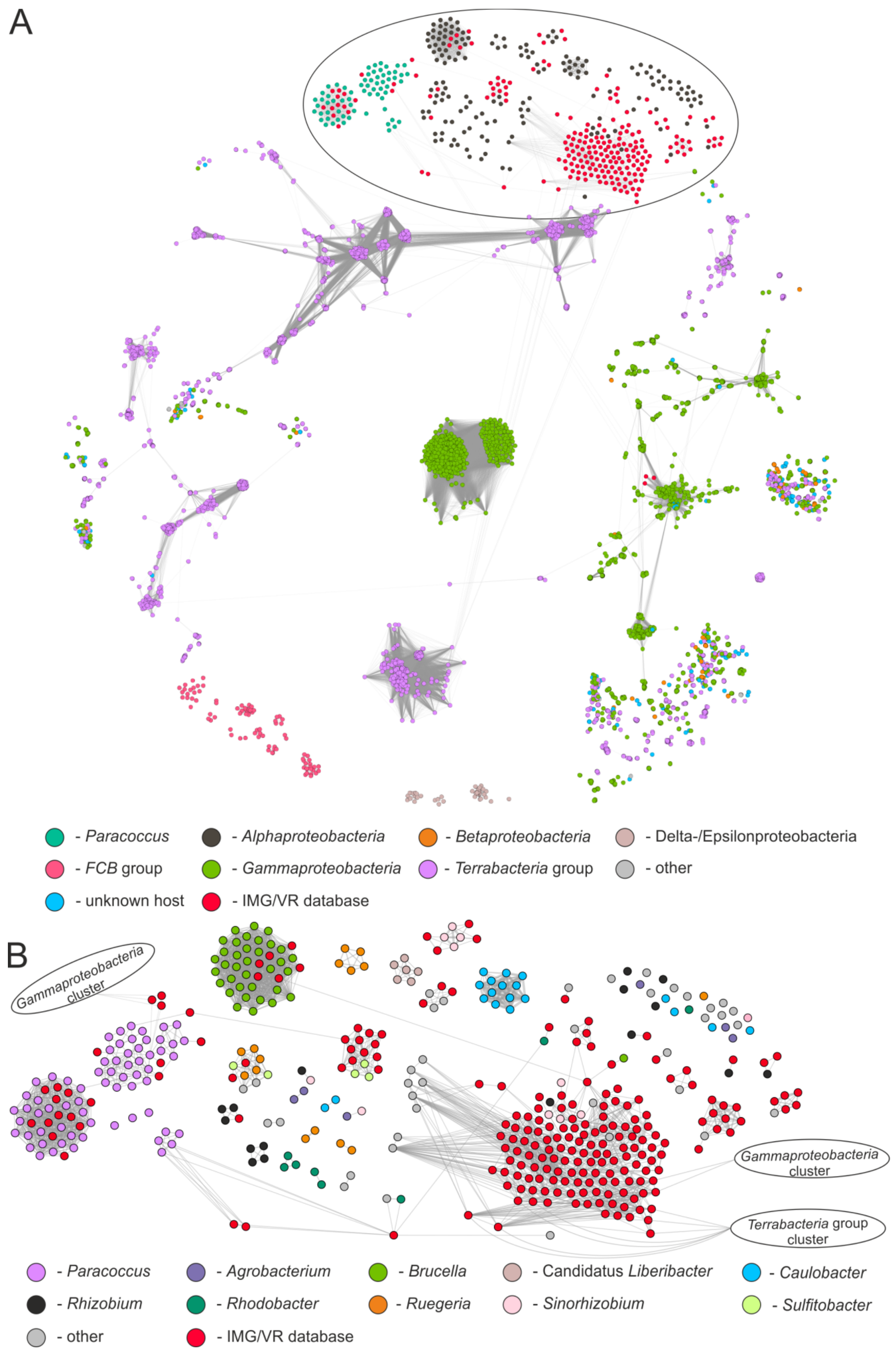

Figure S2. Protein-based similarity network analysis of *Paracoccus* (pro)phages, other bacteriophages retrieved from the NCBI Viruses database and selected viral contigs from the IMG/VR database (for details see *Comparative genomics* section in the manuscript). (**A**) Overall similarity network of known bacterial phages and viral contigs from the IMG/VR database. Nodes are coloured based on the taxonomy of the phage host (at phylum level, except *Proteobacteria* where classes are considered). The nodes for the IMG/VR contigs are presented in red. *Paracoccus* (pro)phages are distinguished within the network. The host taxonomy is based on manually-curated qualifiers in the source section and organism name of the virus GenBank files. (**B**) Magnified image of *Alphaproteobacteria* (pro)phage network and IMG/VR viral contigs. Outgroups connected with the main *Alphaproteobacteria* network are indicated with ellipses.

Table S1. Genes distinguished within the genomes of the vB_PbeS_Pben1, vB_PkoS_Pkon1, vB_PsuS_Psul1, vB_PthS_Pthi1 and vB_PyeM_Pyei1 phages.

Gene	Coding region (bp)	Strand	Protein size (aa)	Putative function
**vB_PbeS_Pben1 of *P. bengalensis* DSM 17099**				
pben1_p01	158-232	→	-	tRNA$_{Val}$(TAC)
pben1_p02	236-1381	←	381	integrase; tyrosine recombinase XerC/XerD
pben1_p03	1371-1643	←	90	hypothetical protein
pben1_p04	1687-1938	←	83	hypothetical protein
pben1_p05	1931-2182	←	83	hypothetical protein
pben1_p06	2182-2889	←	235	hypothetical protein
pben1_p07	2889-3176	←	95	hypothetical protein
pben1_p08	3169-3336	←	55	hypothetical protein
pben1_p09	3329-3724	←	131	hypothetical protein
pben1_p10	3721-4152	←	143	hypothetical protein
pben1_p11	4149-4436	←	95	hypothetical protein
pben1_p12	4503-5354	←	283	hypothetical protein
pben1_p13	5382-5708	←	108	hypothetical protein
pben1_p14	5712-5912	←	66	hypothetical protein
pben1_p15	5909-6106	←	65	hypothetical protein
pben1_p16	6090-6254	←	54	hypothetical protein
pben1_p17	6251-6643	←	130	hypothetical protein
pben1_p18	6640-6906	←	88	hypothetical protein
pben1_p19	7134-7472	←	112	hypothetical protein
pben1_p20	7475-7837	←	120	CI-like repressor
pben1_p21	7935-8183	→	82	Cro-like protein
pben1_p22	8180-8533	→	117	hypothetical protein
pben1_p23	8533-8685	→	50	hypothetical protein
pben1_p24	8682-9038	→	118	hypothetical protein
pben1_p25	9056-9463	←	135	type II toxin-antitoxin, HicB family antitoxin
pben1_p26	9476-9664	←	62	type II toxin-antitoxin, HicA family toxin
pben1_p27	10058-10291	→	77	hypothetical protein
pben1_p28	9845-10075	←	76	hypothetical protein
pben1_p29	10288-10989	→	233	adenine DNA methyltransferase
pben1_p30	10986-11183	→	65	hypothetical protein
pben1_p31	11185-11415	→	76	hypothetical protein
pben1_p32	11399-12019	→	206	putative phage replication protein
pben1_p33	12266-12835	→	189	hypothetical protein
pben1_p34	12835-13476	→	213	hypothetical protein
pben1_p35	13642-14346	→	240	hypothetical protein
pben1_p36	14481-15167	→	228	terminase small subunit
pben1_p37	15182-17362	→	726	terminase large subunit
pben1_p38	17362-17562	→	66	hypothetical protein
pben1_p39	17564-19063	→	499	portal protein
pben1_p40	19056-21137	→	693	putative phage protease and major capsid protein
pben1_p41	21222-21572	→	116	hypothetical protein
pben1_p42	21572-21895	→	107	hypothetical protein
pben1_p43	21898-22320	→	140	hypothetical protein
pben1_p44	22345-22764	→	139	tail protein
pben1_p45	22767-23102	→	111	hypothetical protein
pben1_p46	23192-23413	→	73	hypothetical protein
pben1_p47	23838-26030	→	730	tail tape measure protein
pben1_p48	26027-26683	→	218	hypothetical protein
pben1_p49	26680-27267	→	195	hypothetical protein
pben1_p50	27539-27787	→	82	hypothetical protein
pben1_p51	27256-27609	←	117	hypothetical protein
pben1_p52	27806-27991	→	61	hypothetical protein
pben1_p53	28040-28261	→	73	hypothetical protein
pben1_p54	28267-28686	→	139	hypothetical protein
pben1_p55	28673-30850	→	725	tail fiber protein
pben1_p56	30862-31506	→	214	putative phage holin
pben1_p57	31503-33698	→	731	tail fiber protein
pben1_p58	33703-34491	→	262	hypothetical protein
pben1_p59	34566-34940	→	124	hypothetical protein
pben1_p60	34933-35031	→	32	hypothetical protein

pben1_p61	35028-35234	→	68	hypothetical protein
pben1_p62	35243-35503	→	86	hypothetical protein
pben1_p63	35503-36336	→	277	hypothetical protein
pben1_p64	36360-36566	←	68	hypothetical protein
pben1_p65	36625-37044	←	139	hypothetical protein
pben1_p66	37193-37228	←	32	hypothetical protein
pben1_p67	37269-38093	→	274	peptidoglycan-binding protein; lysozyme
pben1_p68	38097-38297	→	66	hypothetical protein
pben1_p69	38297-38425	→	42	hypothetical protein
pben1_p70	38516-39031	←	171	hypothetical protein
pben1_p71	39117-39242	→	41	hypothetical protein
pben1_p72	39545-39766	→	73	hypothetical protein
**vB_PkoS_Pkon1 of *P. kondratievae* NCIMB 13773T**				
pkon1_p01	63-1070	←	335	integrase; tyrosine recombinase XerC/XerD
pkon1_p02	1067-1231	←	54	hypothetical protein
pkon1_p03	1359-1610	←	83	hypothetical protein
pkon1_p04	1610-1792	←	60	hypothetical protein
pkon1_p05	1792-1983	←	63	hypothetical protein
pkon1_p06	1976-2437	←	153	hypothetical protein
pkon1_p07	2427-3011	←	194	hypothetical protein
pkon1_p08	2998-3342	←	114	hypothetical protein
pkon1_p09	3479-3727	←	82	hypothetical protein
pkon1_p10	3730-4113	←	127	HNHc endonuclease
pkon1_p11	4110-4610	←	166	hypothetical protein
pkon1_p12	4610-4816	←	68	hypothetical protein
pkon1_p13	5028-5513	←	161	single-stranded DNA-binding protein (SSB)
pkon1_p14	5513-6094	←	193	essential recombination function protein (ERF)
pkon1_p15	6091-6309	←	72	hypothetical protein
pkon1_p16	6306-6671	←	121	cysteine-rich zinc finger protein
pkon1_p17	6673-6882	←	69	hypothetical protein
pkon1_p18	6879-7058	←	59	hypothetical protein
pkon1_p19	7079-7291	←	70	hypothetical protein
pkon1_p20	7385-7627	←	80	hypothetical protein
pkon1_p21	8455-9183	←	242	hypothetical protein
pkon1_p22	9186-9389	←	67	hypothetical protein
pkon1_p23	9563-9874	←	103	CI-like repressor
pkon1_p24	9948-10244	→	98	Cro-like protein
pkon1_p25	10241-10450	→	69	hypothetical protein
pkon1_p26	10447-10704	→	85	hypothetical protein
pkon1_p27	10706-11158	→	150	RusA-like crossover junction endodeoxyribonuclease
pkon1_p28	11207-12592	→	461	DnaB replicative DNA helicase
pkon1_p29	12596-13303	→	235	hypothetical protein
pkon1_p30	13300-13797	→	165	DNA binding protein
pkon1_p31	13794-14009	→	71	NrdH-redoxin; thioredoxin (TRX)-like superfamily
pkon1_p32	14140-14796	→	218	hypothetical protein
pkon1_p33	15230-15745	→	171	terminase small subunit
pkon1_p34	15717-17180	→	487	terminase large subunit
pkon1_p35	17190-18599	→	469	portal protein
pkon1_p36	18611-19357	→	248	structural protein
pkon1_p37	19454-20443	→	329	major capsid protein
pkon1_p38	20436-21302	→	288	hypothetical protein
pkon1_p39	21302-21502	→	66	hypothetical protein
pkon1_p40	21502-21918	→	138	hypothetical protein
pkon1_p41	21918-22268	→	116	hypothetical protein
pkon1_p42	22476-23507	→	343	head morphogenesis
pkon1_p43	23508-23849	←	113	type II toxin-antitoxin system HipB-like antitoxin
pkon1_p44	23862-24113	←	83	type II toxin-antitoxin system RelE-family toxin
pkon1_p45	24188-24604	→	138	tail protein
pkon1_p46	24601-25029	→	142	tail terminator
pkon1_p47	25051-25503	→	150	hypothetical protein
pkon1_p48	25506-26042	→	178	hypothetical protein
pkon1_p49	25898-26311	→	137	hypothetical protein
pkon1_p50	26390-27367	→	325	hypothetical protein
pkon1_p51	27471-27728	←	85	Arc-like DNA-binding domain
pkon1_p52	27825-28016	→	63	Arc-family transcriptional repressor
pkon1_p53	28060-28845	→	261	DNA-binding domain (KilA-N) containing protein
pkon1_p54	28842-29135	→	97	hypothetical protein

pkon1_p55	29210-29635	→	141	hypothetical protein
pkon1_p56	29757-30770	→	337	hypothetical protein
pkon1_p57	30814-31344	←	176	peptidoglycan binding domain-containing protein
pkon1_p58	31367-33865	→	832	tail length tape measure protein
pkon1_p59	33865-34500	→	211	hypothetical protein
pkon1_p60	34503-35120	→	205	hypothetical protein
pkon1_p61	35125-35322	←	84	hypothetical protein
pkon1_p62	35294-35548	→	65	hypothetical protein
pkon1_p63	35641-35898	→	85	hypothetical protein
pkon1_p64	35895-36323	→	142	hypothetical protein
pkon1_p65	36327-41894	→	1855	tail protein
pkon1_p66	41891-42316	→	141	tail assembly chaperone protein
pkon1_p67	42338-42529	→	63	hypothetical protein
pkon1_p68	42576-42749	→	57	hypothetical protein
pkon1_p69	42948-43583	→	211	hypothetical protein
pkon1_p70	43815-44135	→	106	LydA holin superfamily III
pkon1_p71	44143-44355	←	70	XRE family transcriptional regulator
pkon1_p72	44352-44540	←	62	hypothetical protein
pkon1_p73	44681-45313	→	210	N-acetylmuramoyl-L-alanine amidase; peptidoglycan recognition protein
pkon1_p74	45325-45720	→	131	hypothetical protein
pkon1_p75	45985-46842	←	285	adenine DNA methyltransferase
pkon1_p76	46835-48154	←	439	hypothetical protein
pkon1_p77	48359-48919	←	186	hypothetical protein
pkon1_p78	48912-49097	←	61	hypothetical protein
pkon1_p79	49354-49617	←	87	hypothetical protein
**vB_PsuS_Psul1 of *P. sulfuroxidans* JCM 14013T**				
psul1_p01	77-1165	←	362	integrase; tyrosine recombinase XerC/XerD
psul1_p02	1149-1346	←	65	hypothetical protein
psul1_p03	1343-2026	←	227	hypothetical protein
psul1_p04	2026-2253	←	75	hypothetical protein
psul1_p05	2250-2507	←	85	hypothetical protein
psul1_p06	2507-3661	←	384	DNA polymerase III, beta subunit
psul1_p07	3661-4050	←	129	hypothetical protein
psul1_p08	4141-4386	←	81	hypothetical protein
psul1_p09	4383-4622	←	79	hypothetical protein
psul1_p10	4691-4867	→	58	hypothetical protein
psul1_p11	4864-5073	→	69	hypothetical protein
psul1_p12	5265-5510	←	81	hypothetical protein
psul1_p13	5642-6127	←	161	hypothetical protein
psul1_p14	6129-6578	←	149	hypothetical protein
psul1_p15	6680-6901	←	73	hypothetical protein
psul1_p16	6898-7488	←	196	hypothetical protein
psul1_p17	7650-8042	←	130	CI-like repressor
psul1_p18	8129-8344	→	71	Cro-like protein
psul1_p19	8425-8694	→	89	hypothetical protein
psul1_p20	8694-8846	→	50	hypothetical protein
psul1_p21	8843-9322	→	159	putative phage replication protein
psul1_p22	9319-9957	→	212	hypothetical protein
psul1_p23	10124-10696	→	190	hypothetical protein
psul1_p24	10693-11412	→	239	hypothetical protein
psul1_p25	11501-12220	→	239	hypothetical protein
psul1_p26	12313-13011	→	232	terminase small subunit
psul1_p27	13022-15211	→	729	terminase large subunit
psul1_p28	15211-15408	→	65	head-tail connector
psul1_p29	15410-16891	→	493	portal protein
psul1_p30	16888-18987	→	669	putative phage protease and major capsid protein
psul1_p31	19068-19418	→	116	scaffolding protein
psul1_p32	19418-19756	→	112	hypothetical protein
psul1_p33	19753-20175	→	140	hypothetical protein
psul1_p34	20199-20618	→	139	tail protein
psul1_p35	20621-20977	→	118	hypothetical protein
psul1_p36	21073-21303	→	76	hypothetical protein
psul1_p37	21319-23601	→	760	tail tape measure protein
psul1_p38	23603-24238	→	211	hypothetical protein
psul1_p39	24235-24852	→	205	hypothetical protein
psul1_p40	24927-25361	→	144	hypothetical protein

psul1_p41	25365-28796	→	1143	tail fiber protein
psul1_p42	28793-29377	→	194	hypothetical protein
psul1_p43	29736-30026	→	96	hypothetical protein
psul1_p44	30287-30550	→	87	hypothetical protein
psul1_p45	30798-31337	→	179	lysozyme
psul1_p46	31339-31578	→	79	hypothetical protein
psul1_p47	31571-31738	←	55	hypothetical protein
psul1_p48	32013-32210	←	65	hypothetical protein
psul1_p49	32391-32666	←	91	hypothetical protein
psul1_p50	32785-32991	←	68	cold shock protein CspA
psul1_p51	34420-34428	→	52	hypothetical protein
psul1_p52	34904-35281	←	126	hypothetical protein
psul1_p53	35287-35409	←	41	hypothetical protein
psul1_p54	35406-35579	←	57	hypothetical protein
psul1_p55	36165-36707	←	54	hypothetical protein
psul1_p56	36719-37150	←	147	hypothetical protein
psul1_p57	37147-37575	←	142	hypothetical protein
**vB_PthS_Pthi1 of *P. thiocyanatus* JCM 20756**				
pthi1_p01	42-1142	←	366	integrase; tyrosine recombinase XerC/XerD
pthi1_p02	1118-1348	←	76	excisionase
pthi1_p03	1348-1533	←	61	hypothetical protein
pthi1_p04	1530-1787	←	85	hypothetical protein
pthi1_p05	1780-2565	←	261	hypothetical protein
pthi1_p06	2562-2981	←	139	hypothetical protein
pthi1_p07	2978-5623	←	881	helicase
pthi1_p08	5627-6745[	←	372	DNA polymerase III, beta subunit
pthi1_p09	6880-7029	←	49	hypothetical protein
pthi1_p10	7026-7286	←	86	hypothetical protein
pthi1_p11	7286-7471	←	61	hypothetical protein
pthi1_p12	7707-8018	→	103	CI-like repressor
pthi1_p13	8118-8414	→	98	Cro-like protein
pthi1_p14	8431-8709	→	92	hypothetical protein
pthi1_p15	8709-8807	→	32	hypothetical protein
pthi1_p16	8808-8969	→	53	hypothetical protein
pthi1_p17	9020-9304	→	94	hypothetical protein
pthi1_p18	9297-9620	→	52	hypothetical protein
pthi1_p19	9620-10759	→	379	topoisomerase-primase domain containing protein
pthi1_p20	10752-12596	→	614	DNA primase
pthi1_p21	12578-12766	→	62	hypothetical protein
pthi1_p22	12813-13058	→	81	hypothetical protein
pthi1_p23	13055-13813	→	252	hypothetical protein
pthi1_p24	13995-14345	→	116	HNH endonuclease
pthi1_p25	14532-15014	→	160	hypothetical protein
pthi1_p26	15001-16707	→	568	terminase large subunit
pthi1_p27	16709-17950	→	413	portal protein
pthi1_p28	17952-18818	→	288	Clp protease; head maturation protease
pthi1_p29	18820-20034	→	404	major capsid protein
pthi1_p30	20097-20699	→	200	hypothetical protein
pthi1_p31	20696-21055	→	119	head-tail connector protein
pthi1_p32	21057-21383	→	108	head-tail adaptor protein
pthi1_p33	21383-21949	→	188	tail-component protein
pthi1_p34	21953-22339	→	128	hypothetical protein
pthi1_p35	22332-22799	→	155	tail protein
pthi1_p36	22809-23141	→	110	structural protein
pthi1_p37	23138-23431	→	97	hypothetical protein
pthi1_p38	23421-23750	←	109	hypothetical protein
pthi1_p39	23773-25725	→	650	tail length tape measure protein
pthi1_p40	25725-26387	→	220	hypothetical protein
pthi1_p41	26387-26977	→	196	hypothetical protein
pthi1_p42	26959-27360	→	133	hypothetical protein
pthi1_p43	27360-29450	→	696	tail fiber protein
pthi1_p44	29515-33924	→	1469	hypothetical protein
pthi1_p45	34148-35119	→	323	hypothetical protein
pthi1_p46	35197-35523	→	108	putative phage LydA holin
pthi1_p47	35767-36186	←	139	hypothetical protein
pthi1_p48	36411-37043	→	210	N-acetylmuramoyl-L-alanine amidase; peptidoglycan recognition protein

pthi1_p49	37054-37449	→	131	hypothetical protein
pthi1_p50	37565-38767	→	400	DNA polymerase IV; UmuC-like DNA-repair protein
pthi1_p51	38839-39159	→	106	hypothetical protein
pthi1_p52	39183-39455	→	90	hypothetical protein
		**vB_PyeM_Pyei1 of *P. yeei* CCUG 32053**		
pyei_p01	60-1166	←	368	integrase; tyrosine recombinase XerC/XerD
pyei_p02	1159-1335	←	58	hypothetical protein
pyei_p03	1335-1718	←	127	hypothetical protein
pyei_p04	1715-1993	←	92	hypothetical protein
pyei_p05	1990-4011	←	673	DNA (cytosine-5-)-methyltransferase
pyei_p06	4125-4283	→	52	chaperone protein DnaJ
pyei_p07	4292-5035	←	247	EaA-like protein
pyei_p08	5032-6708	←	558	helicase
pyei_p09	6705-7070	←	121	endonuclease
pyei_p10	7074-8183	←	369	DNA polymerase III beta subunit
pyei_p11	8174-8674	←	166	hypothetical protein
pyei_p12	8857-9012	←	51	hypothetical protein
pyei_p13	9012-9446	←	144	tellurite resistance protein TerB
pyei_p14	9595-9714	←	39	hypothetical protein
pyei_p15	9714-10004	←	96	hypothetical protein
pyei_p16	10695-10925	←	76	hypothetical protein
pyei_p17	10922-11164	←	80	hypothetical protein
pyei_p18	11161-11379	←	72	hypothetical protein
pyei_p19	11440-12138	←	232	CI-like repressor
pyei_p20	12190-12387	→	65	hypothetical protein
pyei_p21	12387-12551	→	54	hypothetical protein
pyei_p22	12548-13126	→	192	hypothetical protein
pyei_p23	13123-14067	→	314	replication initiation protein
pyei_p24	14120-14833	→	237	DNA-binding protein, antirepressor protein
pyei_p25	14833-15093	→	86	hypothetical protein
pyei_p26	15086-15541	→	151	VRR-NUC domain-containing protein (nuclease)
pyei_p27	15538-16017	→	159	hypothetical protein
pyei_p28	16014-16262	→	82	chaperone protein DnaJ
pyei_p29	16252-16899	→	215	hypothetical protein
pyei_p30	17187-18419	→	410	signal transduction histidine kinase
pyei_p31	18918-19505	→	195	hypothetical protein
pyei_p32	19682-20257	→	191	terminase small subunit
pyei_p33	20226-22076	→	616	terminase large subunit
pyei_p34	22073-23701	→	542	portal protein
pyei_p35	23691-23933	→	80	head completion protein
pyei_p36	23948-24943	→	331	Clp protease; head maturation protease
pyei_p37	24956-25324	→	122	head decoration protein D
pyei_p38	25328-26365	→	345	major capsid protein
pyei_p39	26430-26555	→	41	hypothetical protein
pyei_p40	26601-26990	→	129	hypothetical protein
pyei_p41	26991-27725	→	244	hypothetical protein
pyei_p42	27718-28239	→	173	baseplate assembly protein V
pyei_p43	28250-28414	→	54	hypothetical protein
pyei_p44	28419-28742	→	107	baseplate assembly protein W
pyei_p45	28739-29629	→	296	baseplate assembly protein J
pyei_p46	29633-30190	→	185	tail protein
pyei_p47	30190-31164	→	324	tail protein
pyei_p48	31230-32414	→	394	tail sheath monomer protein
pyei_p49	32427-32942	→	171	tail tube protein
pyei_p50	32939-33247	→	102	tail assembly chaperone protein E
pyei_p51	33247-33369	→	40	hypothetical protein
pyei_p52	33366-33941	←	191	hypothetical protein
pyei_p53	33996-34412	←	138	Arc-like DNA-binding domain-containing protein
pyei_p54	34517-34693	→	58	Arc-family DNA-binding protein
pyei_p55	34767-35591	→	274	DNA-binding protein, antirepressor protein
pyei_p56	35588-35737	→	49	hypothetical protein
pyei_p57	35734-36075	→	113	hypothetical protein
pyei_p58	36136-38625	→	829	tail length tape-measure protein
pyei_p59	38625-39068	→	147	tail protein U
pyei_p60	39065-39274	→	69	tail protein X
pyei_p61	39294-40262	→	322	tail protein D
pyei_p62	40259-40612	→	117	structural protein

pyei_p63	40616-41557	→	313	hypothetical protein
pyei_p64	41618-41950	→	110	hypothetical protein
pyei_p65	41950-43944	→	664	tail fiber protein
pyei_p66	43944-44798	→	284	hypothetical protein
pyei_p67	44930-45196	→	88	hypothetical protein
pyei_p68	45193-45372	→	59	hypothetical protein
pyei_p69	45363-45758	→	131	hypothetical protein
pyei_p70	45763-45957	←	64	hypothetical protein
pyei_p71	46040-47185	→	381	Ca^{2+} ion binding domain containing hemolysin-type protein
pyei_p72	47187-48008	→	273	peptidoglycan-binding protein
pyei_p73	48010-48378	→	122	hypothetical protein
pyei_p74	48375-48845	→	156	structural protein
pyei_p75	49435-50052	→	205	SOS response associated peptidase (SRAP)

Table S2. Reciprocal identities (%) of proteins encoded within the vB_PbeS_Pben1, vB_PkoS_Pkon1, vB_PsuS_Psul1, vB_PthS_Pthi1 and vB_PyeM_Pyei1 phages. Thresholds of e-value 1e-10 and query coverage per HSP of 50% were applied.

Protein (gene) no. within the particular phage	vB_PbeS_Pben1	vB_PkoS_Pkon1	vB_PsuS_Psul1	vB_PthS_Pthi1	vB_PyeM_Pyei1
**1**		pben1_p02 (29%)<br>psul1_p01 (27%)	pben1_p02 (29%)<br>pkon1_p01 (27%)<br>pyei1_p01 (28%)	-	psul1_p01 (28%)
**2**	pkon1_p01 (29%)<br>psul1_p01 (29%)	-	-	-	-
**3**	-	-	-	pben1_p08 (79%)	pkon1_p09 (82%)<br>psul1_p07 (66%)
**4**	-	-	-	-	-
**5**	-	-	-	-	-
**6**	-	-	pthi1_p08 (45%)<br>pyei1_p10 (47%)	psul1_p57 (71%)	-
**7**	-	-	pkon1_p09 (79%)<br>pyei1_p03 (66%)	-	-
**8**	pthi1_p03 (79%)	-	-	psul1_p06 (45%)<br>pyei1_p10 (54%)	-
**9**	-	psul1_p07 (79%)<br>pyei1_p03 (82%)	-	-	-
**10**	-	-	-	-	psul1_p06 (47%)<br>pthi1_p08 (54%)
**11**	-	-	pben1_p65 (65%)<br>pyei1_p70 (56%)	Ppkon1_p06 (89%)<br>Ppsul1_p03 (81%)	-
**12**	-	-	-	-	-
**13**	-	-	-	-	-
**14**	-	-	-	-	-
**15**	-	-	pyei1_p18 (60%)	-	pben1_p17 (56%)
**16**	-	-	-	-	-
**17**	-	-	-	-	-
**18**	-	-	-	-	psul1_p15 (60%)
**19**	-	-	pben1_p22 (94%)	-	-
**20**	-	-	pben1_p23 (73%)	-	-
**21**	-	-	-	-	-
**22**	psul1_p19 (94%)	-	-	-	-
**23**	psul1_p20 (73%)	-	pben1_p33 (29%)	psul1_p24 (44%)	-
**24**	-	-	pthi1_p23 (41%)	-	-
**25**	-	-	pben1_p35 (70%)	-	-
**26**	-	-	pben1_p36 (72%)	-	-
**27**	-	-	pben1_p37 (79%)<br>pyei1_p33 (24%)	-	-
**28**	-	-	pben1_p38 (69%)	pyei1_p36 (33%)	-
**29**	-	-	pben1_p39 (73%)	-	pkon1_p32 (38%)
**30**	-	-	pben1_p40 (72%)	-	-
**31**	-	-	pben1_p41 (80%)	-	-
**32**	-	pyei1_p29 (38%)	pben1_p42 (63%)	-	-
**33**	psul1_p23 (29%)	-	pben1_p43 (63%)	-	pben1_p37 (24%)<br>psul1_p27 (24%)
**34**	-	-	pben1_p44 (68%)<br>pthi1_p35 (28%)	-	-
**35**	psul1_p25 (70%)	-	pben1_p45 (50%)	pben1_p44 (27%)<br>psul1_p34 (28%)	-
**36**	psul1_p26 (72%)	-	pben1_p46 (49%)	-	pben1_p40 (46%)<br>psul1_p30 (49%)<br>pthi1_p28 (33%)
**37**	psul1_p27 (79%)<br>pyei1_p33 (24%)	-	pben1_p47 (51%)	-	-
**38**	psul1_p28 (69%)	-	pkon1_p59 (54%)	-	-
**39**	psul1_p29 (73%)	-	pkon1_p60 (64%)	psul1_p37 (35%)	-
**40**	psul1_p30 (72%)	-	pkon1_p64 (73%)	-	-
**41**	psul1_p31 (80%)	-	pkon1_p65 (49%)	-	-

**42**	psul1_p32 (63%)	-	-	-	-
**43**	psul1_p33 (63%)	-	-	pben1_p55 (25%)	-
**44**	psul1_p34 (68%)<br>pthi1_p35 (27%)	-	-	-	-
**45**	psul1_p35 (50%)	-	-	-	-
**46**	psul1_p36 (49%)	-	-	pkon1_p70 (63%)	-
**47**	psul1_p37 (51%)	-	-	pben1_p65 (96%)	-
**48**	-	-	-	pkon1_p73 (92%)	-
**49**	-	-	-	pkon1_p74 (73%)	-
**50**	-	-	-	-	-
**51**	-	-	-	-	-
**52**	-	-	-	-	-
**53**	-	-	-		-
**54**	-	-	-		-
**55**	pthi1_p43 (24%)	-	-		-
**56**	-	-	-		-
**57**	-	-	pthi1_p06 (71%)		-
**58**	-	-			-
**59**	-	psul1_p38 (54%)			-
**60**	-	psul1_p39 (64%)			-
**61**	-	-			-
**62**	-	-			-
**63**	-	-			-
**64**	psul1_p11 (65%)<br>pyei1_p70 (56%)	psul1_p40 (73%)			-
**65**	pthi1_p47 (96%)	psul1_p41 (49%)			-
**66**		-			-
**67**	pyei1_p72 (74%)	-			-
**68**	-	-			-
**69**	-	-			-
**70**	-	pthi1_p46 (63%)			pben1_p64 (56%)<br>psul1_p11 (56%)
**71**	-	-			-
**72**	-	-			pben1_p67 (74%)
**73**		pthi1_p48 (92%)			-
**74**		pthi1_p49 (73%)			-
**75**		-			-
**76**		-			
**77**		-			
**78**		-			
**79**		-			

% of identity is presented in parentheses.

Table S3. Putative attachment sites for analyzed *Paracoccus* prophages.

Host	Phage name	Classification	Integration strategy	Integration site	Attachment site sequence (5'→3')
P. bengalensis DSM 17099	vB_PbeS_Pben1	*Siphoviridae*	tyr	Intergenic region	GCGTCTCGTTTACACTGAGA
P. kondratieve NCINM 13773	vB_PkoS_Pkon1		tyr	Gene encoding OmpR family transcriptional regulator	GTTTCTCAA(G/C)CAT
P. sulfuroxidans JCM 14013	vB_PsuS_Psul1		tyr	tRNA-Trp(CCA)	CGGTCTCCAAAACCGAGGGTCGTGGGTTCGAGTC CCCCAACCCCTGCCAGT
P. thiocyanatus JCM 20756	vB_PthS_Pthi1		tyr	tRNA-Ser(GGA)	CCTCACCGTCCGCCA
P. yeei CCUG32053	vB_PyeM_Pyei1	*Myoviridae*	tyr	tRNA-Pro(TGG)	GACGGTTTTGGGTACCGTAGGCCGGAGGTTCGA ATCCTCTCGCCCCGACCAG
P. marcusii	vB_PmaS_IMEP1	*Siphoviridae*	N/A		
P. marinus	vB_PmaS_Shpa		N/A		
P. aminophilus JCM 7686	vB_PamS_Pami1	*Siphoviridae*	tyr	tRNA-Pro(TGG)	CGGTTTTGGGTACCGTAGGCCGGAGGTTCGAATC CTCTCGCCCCGACCACAATTTAGC
	vB_PamS_Pami2		tyr	intergenic	TCAGCGTCTCCGGC
	vB_PamS_Pami3		tyr	tRNA-Arg(CCG)	GGCGCACC(G/C)AAGA
	vB_PamS_Pami4		tyr	partial lyase protein	TAATGAGAACATCTGGCCG
	vB_PamS_Pami5		tyr	tRNA-Gly(TCC)	AGCCTTCCAAGCTGAATACGTGGGTTCGATTCCC ATCACCCGCTCCAA
	vB_PamS_Pami6		ser	tRNA-Met(CAT)	TCGGGTGGGAAAATGGTAGCGGAGGAGGGTTT
P. aminovorans DSM 8537	vB_PamS_PD1		ser	not identified	not identified
P. aminovorans HPD-2	vB_PamP_PD2	*Podoviridae*	tyr	tRNA-Thr(CTG)	CGAGTCCCTTCACCGGCACCA
	vB_PamS_PD3	*Siphoviridae*	ser	not identified	not identified
P. contaminans RKI	vB_PcoS_PD4		tyr	tRNA-Met(CAT)	TTTGGTTGCGGGGGCAGGATTTGAACCTACGACC TTCAGGTTATGAGGCT
	vB_PcoS_PD5		tyr	tRNA-Met(CAT)	CTAAGCCTTTGATATCTTGTATGAAGATTTGGTT GCGGGGGCAGGATTTGAACCTGCGGCCTTCAGG TTATGAG
	vB_PcoS_PD6		ser	not identified	not identified
	vB_PcoS_PD7		ser	not identified	not identified
P. denitrificans DSM 413	vB_PdeP_PD8	*Podoviridae*	tyr	tRNA-Thr(TGT)	ATGGTGCCGGTTGCAGGAATCGAACCCGCGACC TTCTGATTACAAATCAG
P. denitrificans DSM 415	vB_PdeP_PD9		tyr	tRNA-Thr(TGT)	ATGGCGCCGGTTGCAGGAATCGAACCCGCGACC TTCTGATTACAAATCAG
P. denitrificans ISTOD1	vB_PdeS_PD10	*Siphoviridae*	tyr	tRNA-Arg(TCT)	CTGGCGGGGTCGCCATTCTATCAAAAAAT
P. denitrificans PD1222	vB_PdeS_PD11		tyr	tRNA-Ser(GCT)	GCAGCGGAGACGTGGCCGAGTGGTCGAAGGCAC ACCCCTGCTAAG
	vB_PdeP_PD12	*Podoviridae*	tyr	tRNA-Thr(TGT)	CTGATTTGTAATCAGAAGGTCGCGGGTTCGATTC CTGCAACCGGCGCCAT
P. homiensis DSM 17862	vB_PhoS_PD13	*Siphoviridae*	ser	not identified	not identified
P. pantotrophus DSM 1403	vB_PpaP_PD14	*Podoviridae*	tyr	tRNA-Arg(CTT)	AACCTGCCCCTTAGGAGGGG
P. pantotrophus J46 J46	vB_PpaP_PD15		tyr	putative ompR regulator	GTTCCTGAACCATCTTTACGGCG
P. saliphilus DSM 18447	vB_PsaS_PD16	*Siphoviridae*	tyr	tRNA-Gly(GCC)	TGGAGCGGGCGATGGGATACGATCAGACGA
P. sanguinis 10990	vB_PsaS_PD17		tyr	not identified	not identified
P. sanguinis 39524	vB_PsaS_PD18		tnp	not identified	not identified
	vB_PsaS_PD19		tnp	not identified	not identified
P. sanguinis 4681	vB_PsaS_PD20		ser	not identified	not identified
	vB_PsaS_PD21		tyr	not identified	not identified
P. sanguinis 5503	vB_PsaS_PD22		tyr	tRNA-Phe(GAA)	TGGTGCCCGGAGGCGGATTCGAAC
	vB_PsaS_PD23		ser	not identified	not identified
	vB_PsaS_PD24		tyr	tRNA-Met(CAT)	TTTGGTTGCGGGGATAGGATTTGAACCTATGACC TTCAGGTTATGAGCC
	vB_PsaS_PD25		tyr	not identified	not identified

	vB_PsaS_PD26		tnp	not identified	not identified
P. sanguinis DSM 29303	vB_PsaS_PD27		tnp	not identified	not identified
	vB_PsaS_PD28		tnp	not identified	not identified
	vB_PsaS_PD29		tyr	tRNA-Met(CAT)	GTTTTGTACGCGTTATCAAACGCTTACACGCCGCCCTCCGGGGCGGC
P. sediminis DSM 26170	vB_PseS_PD30		tyr	tRNA-Met(CAT)	GGCTCATAACCTGAAGG(T/C)C(A/G)CAGGTTCAAATCCTG(T/C)CCCCGCAACCAAAAT
P. solventivorans DSM 6637	vB_PsoS_PD31		ser	not identified	not identified
Paracoccus sp. BM15	vB_PspS_PD32		tyr	tRNA-Met(CAT)	TTTTTTGGTTGCGGGGGT(A/T)GGATTTGAACC(T/A)ACGACCTTCAGGTTATGAGCC
	vB_PspS_PD33		tyr	tRNA-Met(CAT)	TTTTGGTTGCGGGGG(T/C)AGGATTTGAACCT(A/G)CGACCTTCAGGTTATGAGCC
Paracoccus sp. CBA4604	vB_PspS_PD34		ser	not identified	not identified
	vB_PspS_PD35		ser	not identified	not identified
	vB_PspS_PD36		tyr	tRNA-Met(CAT)	AGGCTCATAACCTGAAGGTCACAGGTTCAAATCCTGTCCCCGCAACCAAAATAC
Paracoccus sp. J39	vB_PspS_PD37	*Siphoviridae*	tyr	dusA	TGTCC(G/C)(T/G)(C/A)(C/A)AGTCCATCATCGG(G/C)GCAACGGACAGC(A/C)(TG)
Paracoccus sp. N5	vB_PspS_PD38		tyr	tRNA-Pro(TGG)	GGTTTTGGGTACCGTAGGCCGGAGGTTCGAATCCTCTCGCCCCGACCA
Paracoccus sp. S4493	vB_PspS_PD39		tyr	tRNA-Gly(CCC)	TGGAGCGGGTGAAGGGAATCGAACCCTCGTCGTAAGCTTGGGAAGC
	vB_PspS_PD40		tyr	intergenic	GTCCTGCCGGGCCTACCA
Paracoccus sp. SCN 68-21	vB_PspP_PD41	*Podoviridae*	tyr	tRNA-Thr(GGT)	CCCTTGGTAAGGGTGAGGTCGAGAGTTCAATCCTCTCTCACAGCACCA
	vB_PspS_PD42	*Siphoviridae*	tyr	tRNA-Cys(GCA)	GGATTGCAAATCCGTGAAGACCGGTTCGATTCCGGTACCCACCTCCA
	vB_PspP_PD43	*Podoviridae*	tyr	tRNA-Lys(CAA)	CGGATTCAAAATCCGTTTCAGCGATGAGTGCCGGTTCGAGTCCGGCCGCCCCTACCACGCTCTTTC
	vB_PspS_PD44		tyr	tRNA-Gln(TTC)	CCCCTACGGGCTGCCACT
P. sphaerophysae HAMBI 3106	vB_PspS_PD45		tyr	tRNA-Met(CAT)	CTCATAACCTGAAGGTCACAGGTTCAAATCCTGTCCCCGCAACCAAA
P. versutus DSM 582	vB_PveS_PD46		ser	not identified	not identified
P. yeei ATCC BAA-599	vB_PyeS_PD47	*Siphoviridae*	tyr	intergenic	GCGCGCCGCCT
	vB_PyeS_PD48		ser	not identified	not identified
	vB_PyeS_PD49		tyr	tRNA-Met(CAT)	TTTTTGGTTGCGGGGGCAGGATTTGAACCTGCGGCCTTCAGGTTATGAGCC
P. yeei TT13	vB_PyeM_PD50	*Myoviridae*	tyr	tRNA-Pro(TGG)	GACGGTTTTGGGTACCGTAGGCCGGAGGTTCGAATCCTCTCGCCCCGACCAGT(A/-)CT(T/C)AGC(T/-)(A/-)TTCGCC
	vB_PyeS_PD51		tyr	tRNA-Asn(GTT)	GCTGAG(T/C)T(T/-)GGCTCCGGCGGTAGGGATCGAACCTACGACCAATTGATTAACAGTCAACTG(A/C)(G/T)(A/C)TACCG
	vB_PyeS_PD52	*Siphoviridae*	ser	not identified	not identified
	vB_PyeS_PD53		ser	not identified	not identified

Table S4. Characteristics of 66 (pro)phages of *Paracoccus* spp. In integration strategy column, values "tyr", "ser" and "tnp" refer to tyrosine recombinase, serine recombinase and Mu-like transposase, respectively. Information about the status of particular phage proteins, whether they were identified ("present", "fused", their specificity or function) or not ("-") was shown and followed with the corresponding accession number of the protein or locus tag (if accession number is not available).

Host	Phage name	Integration strategy	Genome size (bp)	% GC	No. of genes	Repressor	Cro-like protein	Protease	Major capsid protein	Endolysin	Holin	MTases	RMs	Toxin-antitoxin systems/Heavy metal metabolism genes
P. bengalensis DSM 17099	vB_PbeS_Pben1	tyr	39,879	64.3	73	present (AZV00177)	present (AZV00178)	fused (AZV00197)		lysozyme (AZV00224)	putative (AZV00213)	m^4C/m^6A (AZV00186)	-	type II toxin-antitoxin HicAB family (AZV00182, AZV00183)
P. kondratieve NCINM 13773	vB_PkoS_Pkon1	tyr	49,723	60.7	82	present (AZV00252)	present (AZV00253)	present (AZV00265)	present (AZV00266)	N-acetylmuramyol-L-alanine amidase (AZV00302)	holin superfamily III (AZV00299)	m^4C/m^6A (AZV00304)	-	type II toxin-antitoxin RelBE family (AZV00272, AZV00273)
P. sulfuroxidans JCM 14013	vB_PsuS_Psul1	tyr	37,901	60.9	62	present (AZV00325)	putative (AZV00326)	fused (AZV00338)		lysozyme (AZV00353)	putative (AZV00351)	-	-	-
P. thiocyanatus JCM 20756	vB_PthS_Pthi1	tyr	39,547	63.8	54	putative (AZV00377)	present (AZV00378)	present (AZV00393)	present (AZV00394)	N-acetylmuramoyl-L-alanine amidase (AZV00413)	present (AZV00411)	-	-	-
P. yeei CCUG32053	vB_PyeM_Pyei1	tyr	50,161	65.4	73	present (AZV00436)	-	present (AZV00453)	present (AZV00455)	PGBP (AZV00489)	putative (AZV00490)	m^5C (AZV00422)	-	tellurite resistnace protein TerB (AZV00430)
P. marcusii	vB_PmaS_IMEP1	N/A	38,261	64.7	52	not applicable	not applicable	present (AJD83161)	present (AJD83163)	d-alanyl-d-alanine carboxypeptidase (AJD83154)	-	-	-	-
P. marinus	vB_PmaS_Shpa	N/A	42,093	56.4	56	not applicable	not applicable	present (AKG94516)	present (AKG94517)	N-acetylmuramyol-L-alanine amidase (AKG94538)	-	-	-	-
P. aminophilus JCM 7686	vB_PamS_Pami1	tyr	43,882	61.6	57	present (WP_041527566)	-	fused (WP_020949540)		N-acetylmuramyol-L-alanine amidase (WP_041527568)	putative (WP_020949552)	m^5C (WP_020949520), m^4C (WP_020949563)	-	tellurite resistance protein TerB (WP_020949524)
	vB_PamS_Pami2	tyr	37,658	60.8	51	putative (pami2_p01)	-	present (WP_020949981)	present (WP_020949982)	N-acetylmuramyol-L-alanine amidase (WP_041527644)	-	m^6A (WP_020949973)	-	-
	vB_PamS_Pami3	tyr	35,083	61.9	53	present (WP_041527269)	present (pami3_p41)	present (WP_020949981)	present (WP_020949982)	N-acetylmuramyol-L-alanine amidase (WP_041527801)	-	-	-	-
	vB_PamS_Pami4	tyr	48,068	60.4	63	present (pami4_p52)	-	present (WP_041527304)	present (WP_020950959)	N-acetylmuramyol-L-alanine	-	m^6A (WP_020952168)	-	-

										amidase (WP_041527846)				
	vB_PamS_Pami5	tyr	43,256	61.0	59	putative (pami5_p50)	-	present (WP_020949981)	present (WP_020949982)	N-acetylmuramyol-L-alanine amidase (WP_041527914)	-	m5C (WP_020951349)	-	-
	vB_PamS_Pami6	ser	38,779	61.6	57	-	-	present (WP_041527304)	present (WP_020950959)	N-acetylmuramyol-L-alanine amidase (WP_041527973)	putative (WP_020951603)	m6A (WP_020951613)	-	-
P. aminovorans DSM 8537	vB_PamS_PD1	ser	46,846	66.9	53	-	-	fused (WP_062563780)		muramidase (WP_062563501)	-	m5C (WP_074969511), m4C/m6A (WP_062563481)	-	-
P. aminovorans HPD-2	vB_PamP_PD2	tyr	42,749	63.7	80	present (WP_062562175)	present (pd2_p27)	-	putative (WP_062560358)	chitinase (WP_062560378)	putative (WP_062560376)	-	-	-
	vB_PamS_PD3	ser	41,904	67,7	48	-	-	fused (WP_062563780)		muramidase (WP_062563501)	putative (WP_062563500)	m4C/m6A (WP_062563481), m5C (WP_062563483)	-	-
P. contaminans RKI	vB_PcoS_PD4	tyr	43,069	65.1	47	-	putative (WP_085378704)	fused (WP_085376815)		muramidase (WP_085376825)	putative (WP_085376824)	m4C/m6A (WP_081962489), m5C (WP_081962487), m4C/m6A (WP_085376809)	Type IIG (WP_085376803)	-
	vB_PcoS_PD5	tyr	61,208	65.2	54	present (WP_085377276)	putative (WP_071166986)	fused (WP_085378766)		muramidase (WP_085377247)	-	m5C (pd5_p37), m4C/m6A (WP_085377265)	Type IIG (WP_085377240)	-
	vB_PcoS_PD6	ser	41,102	68,5	50	-	-	present (WP_052512789)	present (WP_043130239)	M15 peptidase (WP_036700213)	-	m4C/m6A (WP_085378397), m5C (WP_085378398)	-	arsenite resistance protein ArsB (WP_043130246)
	vB_PcoS_PD7	ser	51,188	68,2	58	present (WP_085378415)	putative (WP_085378417)	present (WP_085378932)	present (WP_085378441)	M15 peptidase (WP_085378460)	-	m4C/m6A (WP_085378430), m5C (WP_085378431)	-	-
P. denitrificans DSM 413	vB_PdeP_PD8	tyr	43,858	64.6	73	putative (WP_041530394)	-	putative (WP_011749996)	present (WP_011749997)	chitinase (WP_074810605)	putative (WP_011750014)	-	-	-
P. denitrificans DSM 415	vB_PdeP_PD9	tyr	43,779	64.6	73	putative (WP_041530394)	-	putative (WP_011749996)	present (WP_011749997)	chitinase (WP_074988179)	putative (WP_011750014)	-	-	-
P. denitrificans ISTOD1	vB_PdeS_PD10	tyr	41,212	65.1	61	present (WP_1044920	putative (WP_1044920	fused (WP_104492038)		N-acetylmuramyol-	putative (WP_1044920	m5C (WP_104491978)	-	-

						06)	08)			L-alanine amidase (WP_104492072)	70)			
P. denitrificans PD1222	vB_PdeS_PD11	tyr	42,731	64.4	61	putative (WP_041529726)	-	present (WP_011746708)	present (WP_011746709)	N-acetylmuramyol-L-alanine amidase (WP_041529737)	-	m^4C/m^6A (WP_011746688)	-	-
	vB_PdeP_PD12	tyr	43,827	64.6	75	putative (WP_041530394)	putative (WP_041530395)	present (WP_011749996)	present (WP_011749997)	chitinase (WP_011750015)	putative (WP_011750014)	-	-	-
P. homiensis DSM 17862	vB_PhoS_PD13	ser	44,822	67.7	56	-	-	fused (WP_090735001)		muramidase (WP_090735019)	-	m^4C/m^6A (WP_090734988)	-	zinc/cadmium/lead-transporting ATPase ZntA (WP_090735031)
P. pantotrophus DSM 1403	vB_PpaP_PD14	tyr	38,850	62.9	74	putative (WP_072461511)	putative (pd14_p47)	-	present (WP_072461492)	-	putative (WP_072461479)	m^5C (WP_072461501)	-	-
P. pantotrophus J46 J46	vB_PpaP_PD15	tyr	42,547	61.9	72	putative (WP_051419880)	-	-	present (WP_028711062)	N-acetylmuramidase (WP_028711075)	putative (WP_036765989)	m^4C/m^6A (WP_028711054)	-	-
P. saliphilus DSM 18447	vB_PsaS_PD16	tyr	45,275	59.5	55	putative (WP_076526967)	-	fused (WP_076526980)		muramidase (WP_076526997)	putative (WP_076527097)	m^4C/m^6A (WP_076526959)	-	-
P. sanguinis 10990	vB_PsaS_PD17	tyr	52,526	64.2	59	-	putative (WP_052171651)	present (WP_036700379)	present (WP_036700377)	M15 peptidase (WP_036700345)	putative (WP_036700346)	m^4C/m^6A (WP_036700391), m^4C/m^6A (WP_036700393), m^5C (WP_036700407)	type III (WP_036700399, WP_036700401)	-
P. sanguinis 39524	vB_PsaS_PD18	tnp	41,698	68.5	60	present (WP_052176100)	-	present (WP_036730605)	present (WP_036730601)	N-acetylmuramyol-L-alanine amidase (WP_036730626)	-	m^4C/m^6A (WP_052176079.1)	-	-
	vB_PsaS_PD19	tnp	39,502	68.6	53	present (WP_052176219)	-	present (WP_036731616)	present (WP_036731618)	N-acetylmuramyol-L-alanine amidase (WP_036731608)	-	m^4C/m^6A (WP_036731591)	-	-
P. sanguinis 4681	vB_PsaS_PD20	ser	41,827	68.4	48	-	-	present (WP_052512789)	present (WP_043130239)	M15 peptidase (WP_036700213)	putative (WP_036700215)	m^4C/m^6A (WP_043130228), m^5C (WP_043130230)	-	arsenite resistance protein ArsB (WP_043130246)
	vB_PsaS_PD21	tyr	38,594	65.3	46	-	putative (WP_043132459)	fused (WP_043132429)		muramidase (WP_043132468)	putative (WP_043132452)	m^4C/m^6A (IX54_RS07405) , m^5C (WP_043132412), m^4C/m^6A (WP_043132414)	-	-
P. sanguinis 5503	vB_PsaS_PD22	tyr	53,314	66.0	59	-	putative (WP_0367063	present (WP_0367063	present (WP_0521747	N-acetylmuramidas	putative (WP_0367062	m^4C/m^6A (WP_036706381)	type IIG	-

							20)	12)	28)	e (WP_052174723)	68)		(WP_036706248)	
	vB_PsaS_PD23	ser	61,802	66.5	70	-	-	fused (WP_036706671)		muramidase (WP_036706647)	-	m^4C/m^6A (WP_036706686)	type I (WP_036706619, WP_036706622, WP_081962464, WP_036706627)	multidrug efflux system AcrABCR (WP_052174753 - WP_052174754, WP_036706633)
	vB_PsaS_PD24	tyr	49,592	65.1	52	-	putative (WP_036706775)	present (WP_036706810)	present (WP_036706813)	muramidase (WP_036706958)	putative (WP_036706836)	m^4C/m^6A (WP_036706793), m^4C/m^6A (WP_036706795)	type III (WP_052174762, WP_036706790)	type II Ttoxin-antitoxin system RelBE family (WP_036706860, WP_036706862)
	vB_PsaS_PD25	tyr	32,827	66.9	42	-	putative (WP_052174770)	fused (WP_036707013)		M15 peptidase (WP_036706981)	putative (WP_036706982)	m^4C/m^6A (WP_036707033), m^5C (WP_036707038), m^4C/m^6A (pd25_p36)	-	-
	vB_PsaS_PD26	tnp	44,577	68.5	56	present (WP_036707318)	putative (WP_052174792)	present (WP_036707349)	present (WP_036701237)	N-acetylmuramoyl-L-alanine amidase (WP_036707337)	-	m^4C/m^6A (WP_036707315)	-	-
	vB_PsaS_PD27	tnp	41,697	68.5	60	present (WP_052176100)	present (WP_036730680)	present (WP_036730605)	present (WP_036730601)	N-acetylmuramoyl-L-alanine amidase (WP_036730626)	-	m^4C/m^6A (WP_052176079)	-	-
P. sanguinis DSM 29303	vB_PsaS_PD28	tnp	39,255	68.5	57	present (WP_074826995)	-	present (WP_036731616)	present (WP_036731618)	N-acetylmuramoyl-L-alanine amidase (WP_036731608)	-	m^4C/m^6A (WP_036731591)	-	-
	vB_PsaS_PD29	tyr	51,410	65.3	58	-	putative (WP_052176218)	present (WP_036731552)	present (WP_036731556)	muramidase (WP_074827033) and peptidase M15 (WP_036734832)	putative (WP_036734830)	m^4C/m^6A (WP_036731541), m^4C/m^6A (WP_036731536), m^5C (pd29_p48)	type II (WP_036731533, WP_074827039)	-
P. sediminis DSM 26170	vB_PseS_PD30	tyr	48,532	64.7	48	-	putative (WP_089386171)	present (WP_089386151)	present (WP_089386153)	M15 peptidase (WP_089386138)	putative (WP_017998490)	m^4C/m^6A (WP_089386160), m^4C/m^6A (WP_089386161),	type I (WP_089386163,	-

												m5C (WP_089386167)	WP_089386164, WP_089386165)	
P. solventivorans DSM 6637	vB_PsoS_PD31	ser	42,946	66.0	63	putative (WP_073062047)	-	present (WP_073061259)	present (WP_073061262)	muramidase (WP_073061326)	putative (WP_073061329)	-	-	-
Paracoccus sp. BM15	vB_PspS_PD32	tyr	51,828	60.8	51	-	putative (WP_101459025)	present (WP_101459041)	present (WP_101459043)	M15 peptidase (WP_101459061)	-	m^4C/m^6A (WP_101459033)	type I (WP_101459030, WP_101459031, WP_101461804, WP_101459032)	-
	vB_PspS_PD33	tyr	50,035	63.6	58	-	putative (WP_101461915)	present (WP_101459794)	present (WP_101459043)	M15 peptidase (WP_101459809)	-	m^4C/m^6A (WP_101459785), m^5C (WP_101459786)	Type II (WP_101459812,	lead, cadmium, zinc, mercury transporter (WP_101461915), coper transporting ATPase (WP_101461912) multicoper oxidase family protein (WP_101459778)
Paracoccus sp. CBA4604	vB_PspS_PD34	ser	44,369	67.8	53	-	-	fused (WP_101498968)		muramidase (WP_101498972)	-	m^4C/m^6A (WP_101498964)	-	zinc/cadmium/lead-transporting ATPase ZntA (WP_090735031)
	vB_PspS_PD35	ser	50,207	63.2	54	present (WP_101498988)	putative (WP_101498990)	fused (WP_101499007)		M15 peptidase (WP_101500871)	-	m^4C/m^6A (WP_101498999)	-	-
	vB_PspS_PD36	tyr	44,896	64.7	53	-	putative (WP_101498361)	fused (WP_101498348)		M15 peptidase (WP_101500774)	putative (WP_101498335)	m^5C (WP_101498356), m^4C/m^6A (WP_101500775)	type II (WP_101498366, WP_101498367)	-
Paracoccus sp. J39	vB_PspS_PD37	tyr	40,213	63.6	65	present (WP_051476444)	-	present (WP_028716890)	present (WP_028716889)	PGBP-N-acetylmuramidase (WP_028716871)	-	-	-	type II toxin-antitoxin system HicAB family (WP_028716902, WP_028716903)
Paracoccus sp. N5	vB_PspS_PD38	tyr	46,553	64.3	66	putative (WP_017999905)	-	fused (WP_017999920)		muramidase (WP_017999939) and PGBP - N-acetylmuramidase (WP_017999945)	putative (WP_036744603)	-	-	-
Paracoccus sp. S4493	vB_PspS_PD39	tyr	37,866	62.7	55	putative (WP_071209455)	putative (pd39_p12)	present (WP_045980756)	present (WP_052714499)	N-acetylmuramoyl-L-alanine amidase	putative (WP_045980767)	m^4C/m^6A (WP_045980739)	-	-

										(WP_052714511)				
	vB_PspS_PD40	tyr	36,248	63.7	50	putative (WP_0459821 96)	putative (pd40_p36)	present (WP_0459821 85)	present (WP_0459821 84)	N-acetylmuramoyl-L-alanine amidase (WP_071209474)	putative (WP_0459821 72)	m^5C (WP_071209475)	-	-
Paracoccus sp. SCN 68-21	vB_PspP_PD41	tyr	48,266	64.6	63	putative (ODT61018)	-	-	present (ODT60961)	muramidase (ODT61017)	putative (ODT60966)	m^4C/m^6A (ODT60998)	-	-
	vB_PspS_PD42	tyr	42,396	63.7	55	putative (WP_0722954 89)	putative (pd42_p14)	fused (WP_072295500)		N-acetylmuramoyl-L-alanine amidase (WP_072295521)	putative (WP_0722955 22)	m^4C/m^6A (WP_072295480), m^4C/m^6A (WP_072295482), m^4C/m^6A (WP_072295523)	-	-
Paracoccus sp. SM22M-07	vB_PspP_PD43	tyr	44,599	63.4	67	putative (WP_0722961 21)	putative (pd43_p21)	present (WP_0722961 40)	present (WP_0722961 41)	muramidase (WP_072296160)	putative (WP_0722961 58)	m^5C (WP_072296110)	-	-
	vB_PspS_PD44	tyr	41,926	63.9	71	putative (WP_0722977 37)	putative (WP_0722977 38)	present (WP_0722977 49)	present (WP_0722977 50)	N-acetylmuramoyl-L-alanine amidase (WP_072297768)	putative (WP_0722977 69)	-	-	TerC family protein (WP_072297798)
P. sphaerophysae HAMBI 3106	vB_PspS_PD45	tyr	58,117	66.0	59	-	putative (WP_0367179 17)	fused (WP_036717976)		muramidase (WP_036718003)	-	m^4C/m^6A (WP_036717944), m^5C (pd45_p21), m^4C/m^6A (WP_036718022)	-	type II toxin-antitoxin system VapBC family (WP_036718052, WP_036717998)
P. versutus DSM 582	vB_PveS_PD46	ser	41,696	67.6	49	-	-	fused (WP_036751158)		M15 peptidase (WP_036751128)	putative (WP_0367511 31)	m^5C (pd46_p30), m^4C/m^6A (WP_036751173)	-	-
	vB_PyeS_PD47	tyr	56,744	61.8	59	-	putative (WP_0287206 66)	present (WP_0287206 37)	present (WP_0287206 35)	M15 peptidase (WP_028720621)	putative (WP_0287206 20)	m^4C/m^6A (WP_028720653), m^4C/m^6A (WP_028720654), m^5C (WP_028720662)	type III (WP_0 515783 29, WP_02 872065 9)	magnesium and cobalt transporter CorA (WP_028720649)
P. yeei ATCC BAA-599	vB_PyeS_PD48	ser	43,101	68.1	55	-	-	fused (WP_028720226)		muramidase (WP_028720236) and peptidase M15 (pd48_p55)	-	m^4C/m^6A (WP_028720217), m^5C (pd48_p23)	-	type II toxin-antitoxin system VapBC family (WP_028720231, WP_028720232)
	vB_PyeS_PD49	tyr	50,833	65.3	60	-	putative (WP_0287208 74)	present (WP_0367636 09)	present (WP_0287208 93)	M15 peptidase (WP_028720907)	putative (WP_0287209 06)	m^5C (WP_028720878), m^4C/m^6A (WP_028720883), m^4C/m^6A (WP_036763606)	type I (WP_0 287208 80, WP_05 157832 5, WP_02 872088 2)	zinc transporter ZitB (WP_007803328), type II toxin-antitoxin system ParDE family (WP_028720911, WP_028720912)
P. yeei TT13	vB_PyeM_PD50	tyr	54,200	65.0	80	present (ATQ56181)	-	present (ATQ56198)	present (ATQ56200)	PGBP (ATQ56229)	-	m^5C 9ATQ56170), m^4C/m^6A (ATQ56173)	-	type II toxin-antitoxin system HicBA family (ATQ56191, ATQ56192)

vB_PyeS_PD51	tyr	38,463	61.7	39	-	-	fused (ATQ56549)		N-acetylmuramyol-L-alanine amidase (ATQ56566)	putative (ATQ56567)	m^5C (ATQ56541)	-	-
vB_PyeS_PD52	ser	52,363	67.8	61	-	-	present (ATQ56683)	present (ATQ56681)	muramidase (ATQ56670)	-	m^5C (pd52_p36), m^4C/m^6A	-	-
vB_PyeS_PD53	ser	44,067	65,3	58	-	-	fused (ATQ56739)		muramidase (ATQ56729)	-	m^5C (ATQ56748), m^4C/m^6A (ATQ56749)	-	type II toxin-antitoxin system HigBA family (ATQ56767, ATQ56766)
